# Comparative Electronic Structures of the Chiral Helimagnets Cr_1/3_NbS_2_ and Cr_1/3_TaS_2_

**DOI:** 10.1021/acs.chemmater.3c01564

**Published:** 2023-08-16

**Authors:** Lilia S. Xie, Oscar Gonzalez, Kejun Li, Matteo Michiardi, Sergey Gorovikov, Sae Hee Ryu, Shannon S. Fender, Marta Zonno, Na Hyun Jo, Sergey Zhdanovich, Chris Jozwiak, Aaron Bostwick, Samra Husremović, Matthew P. Erodici, Cameron Mollazadeh, Andrea Damascelli, Eli Rotenberg, Yuan Ping, D. Kwabena Bediako

**Affiliations:** † Department of Chemistry, University of California, Berkeley, California 94720, United States; ‡ Department of Physics, University of California, Santa Cruz, California 95064, United States; § Quantum Matter Institute, University of British Columbia, Vancouver, British Columbia V6T 1Z4, Canada; ∥ Department of Physics and Astronomy, University of British Columbia, Vancouver, British Columbia V6T 1Z1, Canada; ⊥ Canadian Light Source, Inc., 44 Innovation Boulevard, Saskatoon, Saskatchewan S7N 2V3, Canada; # Advanced Light Source, Lawrence Berkeley National Laboratory, Berkeley, California 94720, United States; ¶ Department of Physics, University of Michigan, Ann Arbor, Michigan 48109, United States; ∇ Department of Materials Science and Engineering, University of Wisconsin, Madison, Wisconsin 53706, United States; ○ Chemical Sciences Division, Lawrence Berkeley National Laboratory, Berkeley, California 94720, United States

## Abstract

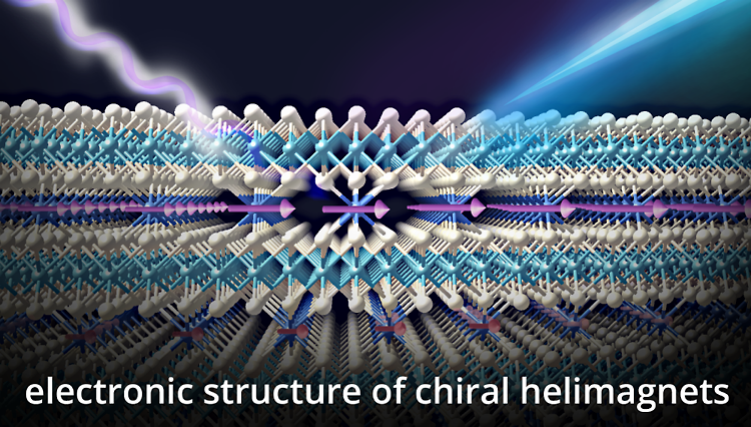

Magnetic materials with noncollinear spin textures are promising for spintronic applications. To realize practical devices, control over the length and energy scales of such spin textures is imperative. The chiral helimagnets Cr_1/3_NbS_2_ and Cr_1/3_TaS_2_ exhibit analogous magnetic-phase diagrams with different real-space periodicities and field dependence, positioning them as model systems for studying the relative strengths of the microscopic mechanisms giving rise to exotic spin textures. Although the electronic structure of the Nb analogue has been experimentally investigated, the Ta analogue has received far less attention. Here, we present a comprehensive suite of electronic structure studies on both Cr_1/3_NbS_2_ and Cr_1/3_TaS_2_ using angle-resolved photoemission spectroscopy and density functional theory. We show that bands in Cr_1/3_TaS_2_ are more dispersive than their counterparts in Cr_1/3_NbS_2_, resulting in markedly different Fermi wavevectors. The fact that their qualitative magnetic phase diagrams are nevertheless identical shows that hybridization between the intercalant and host lattice mediates the magnetic exchange interactions in both of these materials. We ultimately find that ferromagnetic coupling is stronger in Cr_1/3_TaS_2_, but larger spin–orbit coupling (and a stronger Dzyaloshinskii–Moriya interaction) from the heavier host lattice ultimately gives rise to shorter spin textures.

## Introduction

Next-generation spintronic devices utilize the spin degree of freedom to store information.^1,2^ Magnetic materials in which spins order in topologically protected quasiparticles, such as skyrmions or magnetic solitons, are promising platforms for realizing such devices.^3,4^ These chiral spin textures can be manipulated with currents and magnetic fields, which is appealing for various applications in memory, logic, and unconventional computing.^5^ For practical spintronic devices, optimizing the energy and length scales of the spin textures is important: stability at operationally accessible temperatures and fields as well as high density in thin-film architectures is broadly desirable. Strategies to control the microscopic mechanisms that give rise to complex magnetism are thus needed. In terms of materials design, this can be achieved by tailoring the interactions between spin centers as directed by their spatial arrangements and coordination environments.

The chiral helimagnets Cr_1/3_NbS_2_ and Cr_1/3_TaS_2_ are especially well suited for device schemes implementing noncollinear spin textures because of their anisotropic layered structures, which are compatible with thin-film architectures.^6−10^ In these materials, the *S* = 3/2 Cr^3+^ centers occupy pseudo-octahedral sites between layers of 2*H*-NbS_2_ or 2*H*-TaS_2_,^11,12^ forming a  superlattice.^13^ They exhibit easy-plane ferromagnetic (FM) behavior with chiral magnetic ordering out-of-plane: the Cr superlattice breaks the inversion symmetry of the transition metal dichalcogenide (TMD) host lattice along the crystallographic *c*-axis, giving rise to a Dzyaloshinskii–Moriya (DM) interaction, also known as antisymmetric exchange.^14,15^ The DM interaction favors rotation of spins in adjacent Cr layers, which competes with FM exchange to produce one-dimensional helical spin textures that propagate along [001]. Importantly, the application of an in-plane magnetic field creates a chiral soliton lattice (CSL) phase with tunable periodicities up to a critical field, *H*_c_, above which a forced FM (FFM) state is observed.^16−21^ Both Cr_1/3_NbS_2_ and Cr_1/3_TaS_2_ have Curie temperatures, *T*_C_, well above 100 K, and nanoscale soliton wavelengths tunable with fields of 1.5 T or less, thus providing a richly accessible phase space for manipulating chiral spin textures.

Although the magnetic phase diagrams for Cr_1/3_NbS_2_ and Cr_1/3_TaS_2_ are qualitatively analogous, the periodicities and stabilities of their magnetic solitons differ somewhat. Literature reports have established that Cr_1/3_TaS_2_ consistently exhibits a higher *T*_C_,^17,18,21−23^ higher *H*_c_,^18−20,23^ and a shorter soliton wavelength than the Nb analogue.^17,19,21,24^ These observations imply that changing the host lattice from NbS_2_ to TaS_2_ alters the relative strengths of magnetic coupling among Cr centers, manifesting in quantitative changes to their magnetic phase diagrams. However, the origin of magnetic exchange interactions in these materials is still a matter of debate,^25−28^ and comparative studies have been scant.^28^ In particular, the electronic structure of Cr_1/3_TaS_2_ has not been experimentally investigated to date. A detailed study of the electronic structures of both Cr_1/3_NbS_2_ and Cr_1/3_TaS_2_ is thus motivated by the fact that these materials are natural platforms for studying how the length and energy scales of chiral spin textures can be tuned through materials chemistry.

Herein, we present a comprehensive investigation of the electronic structures of Cr_1/3_NbS_2_ and Cr_1/3_TaS_2_ using angle-resolved photoemission spectroscopy (ARPES) and density functional theory (DFT) calculations. We show that the Ta analogue has more dispersive bands, consistent with the greater orbital overlap in the case of Ta. Notably, the Nb and Ta analogues have considerably different Fermi wavevectors, *k*_F_, despite their equivalent magnetic phase diagrams. Using polarization-dependent ARPES, microARPES, and orbital-projected DFT calculations, we assign the parity and orbital character of bands, finding evidence of considerable Cr–host lattice hybridization in the vicinity of the Fermi level, *E*_F_, in both materials. Taken together, these results corroborate existing evidence^18,25−27,29^ that hybridization, rather than the traditionally invoked Ruderman–Kittel–Kasuya–Yosida (RKKY) interaction, drives the magnetic ordering in these materials. We show that although FM coupling is stronger in the Ta analogue, spin–orbit coupling (SOC) is primarily responsible for determining the length scales of spin textures in these materials.

**Figure 1:**
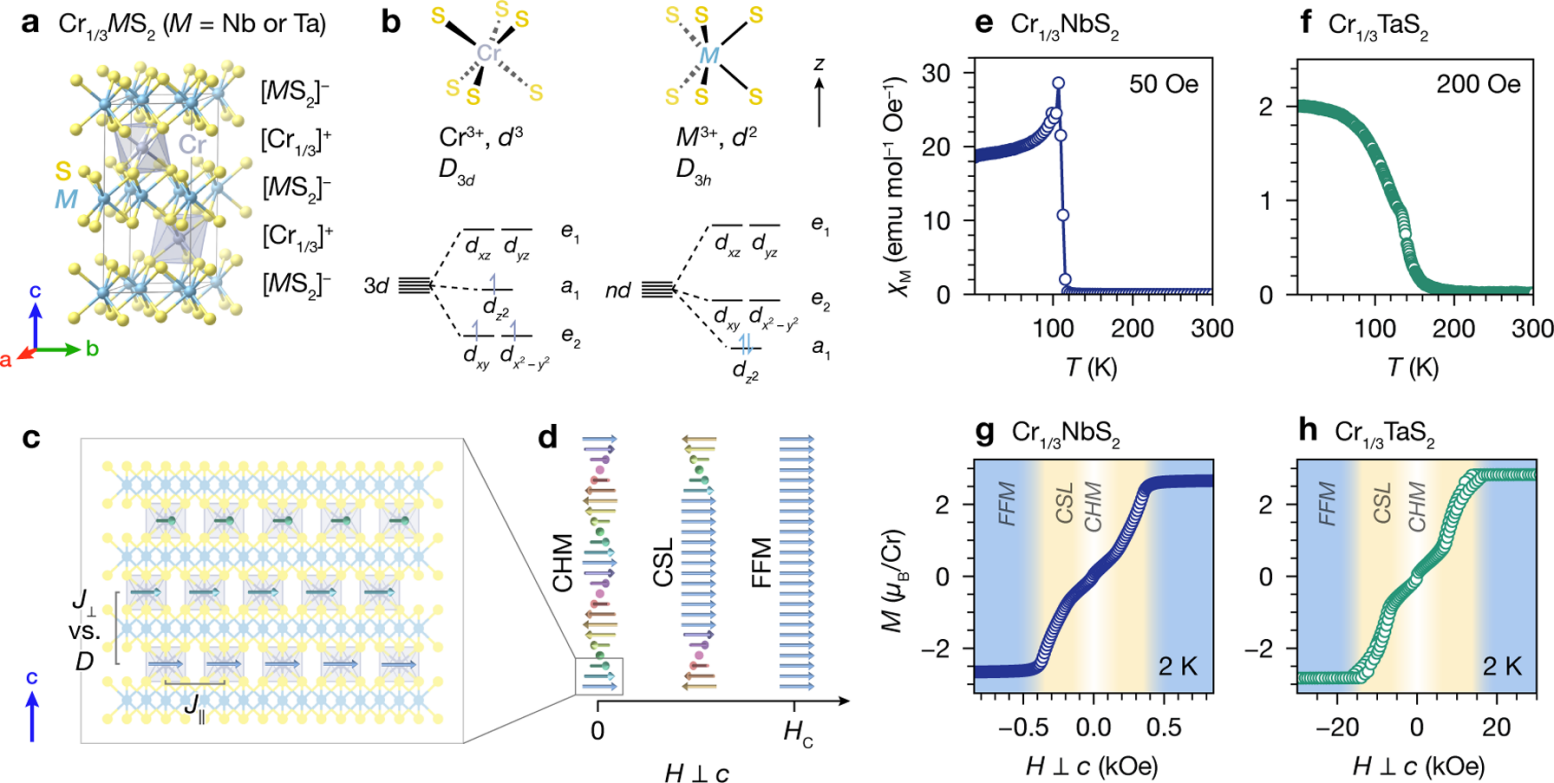
(a) Crystal structure of Cr_1/3_MS_2_, showing formal charges for the MS_2_ and Cr layers from a simple electron-counting picture. (b) Qualitative d-orbital splitting diagrams for isolated Cr and M centers from the local ligand field in Cr_1/3_MS_2_. (c) Schematic illustration of the magnetic structure of Cr_1/3_MS_2_ in the chiral helimagnetic (CHM) state. (d) Schematic representations of spin textures evolving from CHM to CSL to FFM states with increasing applied magnetic field *H* ⊥ *c*. (e) and (f) M(*T*) data for Cr_1/3_NbS_2_ and Cr_1/3_TaS_2_, respectively. (g) and (h) M(*H*) data for Cr_1/3_NbS_2_ and Cr_1/3_TaS_2_, respectively, showing transitions between CHM, CSL, and FFM states.

First, we outline the electronic and magnetic properties of Cr_1/3_MS_2_ (M = Nb or Ta) in brief, as established in the existing literature. According to a simple electron counting scheme, these compounds can be considered as alternating layers of  and . The intercalant layers consist of Cr^3+^ centers occupying 1/3 of the trigonally distorted pseudo-octahedral interstitial sites between layers of 2*H*-MS_2_ (M = Nb or Ta). These intercalant layers donate one electron per formula unit to the MS_2_ host lattice layers (Figure 1a). The qualitative local d-orbital splitting diagrams for the Cr^3+^ (*D*_3*d*_) and M^3+^ (*D*_3*h*_) centers are shown in Figure 1b.^30^ The electronic structure of the periodic solids is more complex, and in reality, the half-filled TMD bands have both  and *d*_*xy*_/ character.^31−33^ Nevertheless, this simplified picture captures (1) charge transfer from the intercalant species to the highest-lying M d bands of the host lattice and (2) the inherently polar nature of these layered intercalation compounds.

The qualitative magnetic properties of Cr_1/3_NbS_2_ and Cr_1/3_TaS_2_ below *T*_C_ are summarized in Figure 1c,d. Within each  layer, the Cr spins exhibit FM coupling through an in-plane exchange constant, *J*_∥_. Between adjacent  layers, FM coupling through an out-of-plane exchange constant, *J*_⊥_, competes energetically with spin canting through a DM interaction term, *D*. At zero field, this results in a continuous helical arrangement of spins, or a CHM ground state, with the magnetic soliton wavelength determined by the ratio of *J*_⊥_ and *D*.^34,35^ With increasing *H* ⊥ *c*, FM regions aligned with the field grow, effectively unwinding the CHM state to create the CSL phase, in which the distance separating adjacent solitons is a function of the magnitude of *H*. Finally, with fields larger than *H*_c_, an FFM state with saturated magnetization is obtained.^16−20,23,36^

In this study, we investigate the electronic structure of Cr_1/3_NbS_2_ and Cr_1/3_TaS_2_ in a comparative context to tease out differences between the two compounds and connect these to their magnetic phase diagrams. To do so, we grew and characterized single crystals, verified their chiral spin textures with magnetometry, carried out a comprehensive suite of ARPES measurements, and conducted DFT band structure calculations, as detailed below.

## Results

### Synthesis, Structure, and Magnetism

Single crystals of Cr_1/3_NbS_2_ and Cr_1/3_TaS_2_ were grown via chemical vapor transport using iodine as a transport agent. X-ray diffraction confirmed that both materials crystallize in the noncentrosymmetric space group *P*6_3_22, with the Cr centers forming a  superlattice (Figure S1 and Tables S1–S3). Cr_1/3_NbS_2_ exhibits a slightly larger in-plane lattice parameter and smaller out-of-plane lattice parameter (*a* = 5.7400(7) Å and *c* = 12.1082(14) Å) compared to Cr_1/3_TaS_2_ (*a* = 5.7155(5) Å and *c* = 12.1751(12) Å). Raman spectroscopy revealed sharp vibrational modes associated with the  superlattices^37^ (Figure S2), and energy-dispersive X-ray spectroscopy indicated Cr/Nb and Cr/Ta ratios of 0.33(1):1 (Figures S3 and S4).

The M(*T*) data show peaks at 110 and 133 K for Cr_1/3_NbS_2_ and Cr_1/3_TaS_2_, respectively, corresponding to the onset of chiral helimagnetism below these temperatures (Figure 1e,f). The metamagnetic transitions across CHM, CSL, and FFM states with applied magnetic field are observed in the M(*H*) data shown in Figure 1g,h, confirming the characteristic spin textures in our samples.^16,19,20^ Both compounds exhibit similar saturation moments (2.7 μ_B_/Cr for Cr_1/3_NbS_2_ and 2.8 μ_B_/Cr for Cr_1/3_TaS_2_), close to the expected spin-only value of 3 μ_B_/Cr. The analogous transitions are observed at fields more than an order of magnitude larger for Cr_1/3_TaS_2_ than Cr_1/3_NbS_2_, with *H*_c_ values of about 0.45 mT for Cr_1/3_NbS_2_ and 16 mT for Cr_1/3_TaS_2_. This is consistent with shorter soliton wavelengths in the Ta analogue.^19−21,38^

### Superlattice Effects on the Electronic Structure

**Figure 2:**
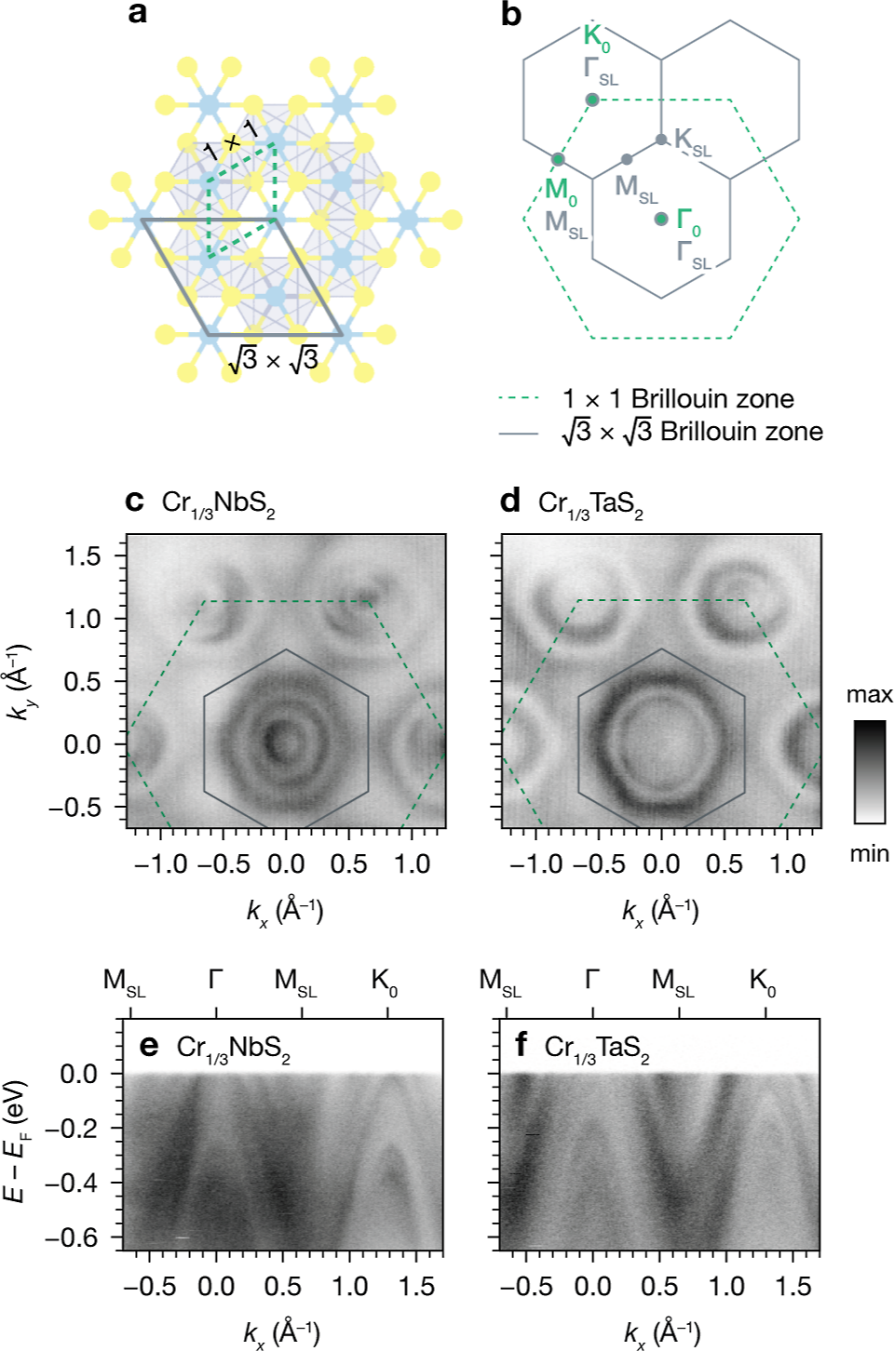
(a) Real-space crystal structure of Cr_1/3_MS_2_ (M = Nb or Ta) viewed along the crystallographic *c*-axis, with overlaid unit cells for the 1 × 1 primitive MS_2_ lattice (dashed green) and the  Cr superlattice (solid gray). (b) Surface Brillouin zones for the 1 × 1 primitive lattice and  superlattice. (c) and (d) ARPES Fermi surfaces of Cr_1/3_NbS_2_ and Cr_1/3_TaS_2_ with the dashed and solid overlaid lines corresponding to the primitive and  superlattice Brillouin zones, respectively. (e) and (f) ARPES band dispersions for Cr_1/3_NbS_2_ and Cr_1/3_TaS_2_ along the Γ–K_0_ direction, showing folding of features from Γ to K_0_ and vice versa (18 K, *h*ν = 79 eV).

After obtaining structural and magnetic evidence of highly ordered  Cr superlattices in Cr_1/3_NbS_2_ and Cr_1/3_TaS_2_, we sought to investigate their influence on the electronic structure of these materials. Figure 2a illustrates the real-space 1 × 1 primitive unit cell for the host lattice TMD and the  superlattice unit cell for the intercalated compounds along [001]. The  unit cell is rotated by 30° compared to the 1 × 1 unit cell. In the reciprocal space, the  superlattice defines a smaller Brillouin zone that is likewise rotated by 30° relative to the primitive Brillouin zone (Figure 2b). To probe the electronic effects of Cr intercalation, we first examined the symmetries of the experimental Fermi surfaces and band dispersions of Cr_1/3_NbS_2_ and Cr_1/3_TaS_2_ using ARPES.

As shown in Figure 2c,d, the Fermi surfaces of both Cr_1/3_NbS_2_ and Cr_1/3_TaS_2_ below *T*_C_ (*h*ν = 79 eV) display multiple nested barrels around Γ and K of the primitive Brillouin zone, which is indicated by the dashed green hexagons. Notably, sixfold symmetry is clearly observed around the primitive K (denoted as K_0_), in contrast with threefold symmetry around K of the host lattice materials 2*H*-NbS_2_ and 2*H*-TaS_2_.^39,40^ Additionally, in the intercalated materials, three-fold symmetry is introduced at K of the  superlattice Brillouin zone (denoted as K_SL_), which is indicated by the solid gray hexagons in Figure 2c,d. Hence, the Fermi surfaces of Cr_1/3_NbS_2_ and Cr_1/3_TaS_2_ display the expected symmetries associated with reconstruction and band folding from the  Cr superlattice.

The ARPES dispersions show clear evidence of band folding as well (Figure 2e,f). Cuts along the Γ–K_0_ direction show the same features at both Γ and K_0_: both materials display several nested hole pockets and parabolic bands below *E*_F_. In contrast, for the host TMDs 2*H*-NbS_2_ and 2*H*-TaS_2_, the bands crossing *E*_F_ have different dispersions and energies at Γ and K. In the Cr-intercalated materials, the  superlattice folds the primitive lattice Γ to K and vice versa, as they both become Γ of the superlattice Brillouin zone (denoted as Γ_SL_ in Figure 2b). Thus, the presence of the same features at both Γ and K_0_ in the ARPES of the Cr-intercalated materials is consistent with  superlattice band folding.

The band folding in the ARPES data reveals that the  Cr superlattice potential is strong in both Cr_1/3_NbS_2_ and Cr_1/3_TaS_2_. Broadly, this electronic reconstruction is in line with previous literature reports on Cr_1/3_NbS_2_,^25,27^ as well as other intercalated TMDs with  transition metal superlattices.^41−44^ The features observed in both materials are qualitatively similar; however, at a glance, the hole pockets in Cr_1/3_TaS_2_ appear to be larger than those found in Cr_1/3_NbS_2_. To contextualize differences in the experimental electronic structures of Cr_1/3_NbS_2_ and Cr_1/3_TaS_2_, we turned to DFT calculations and quantitative analysis of their band dispersions.

### Relative Band Dispersions

**Figure 3:**
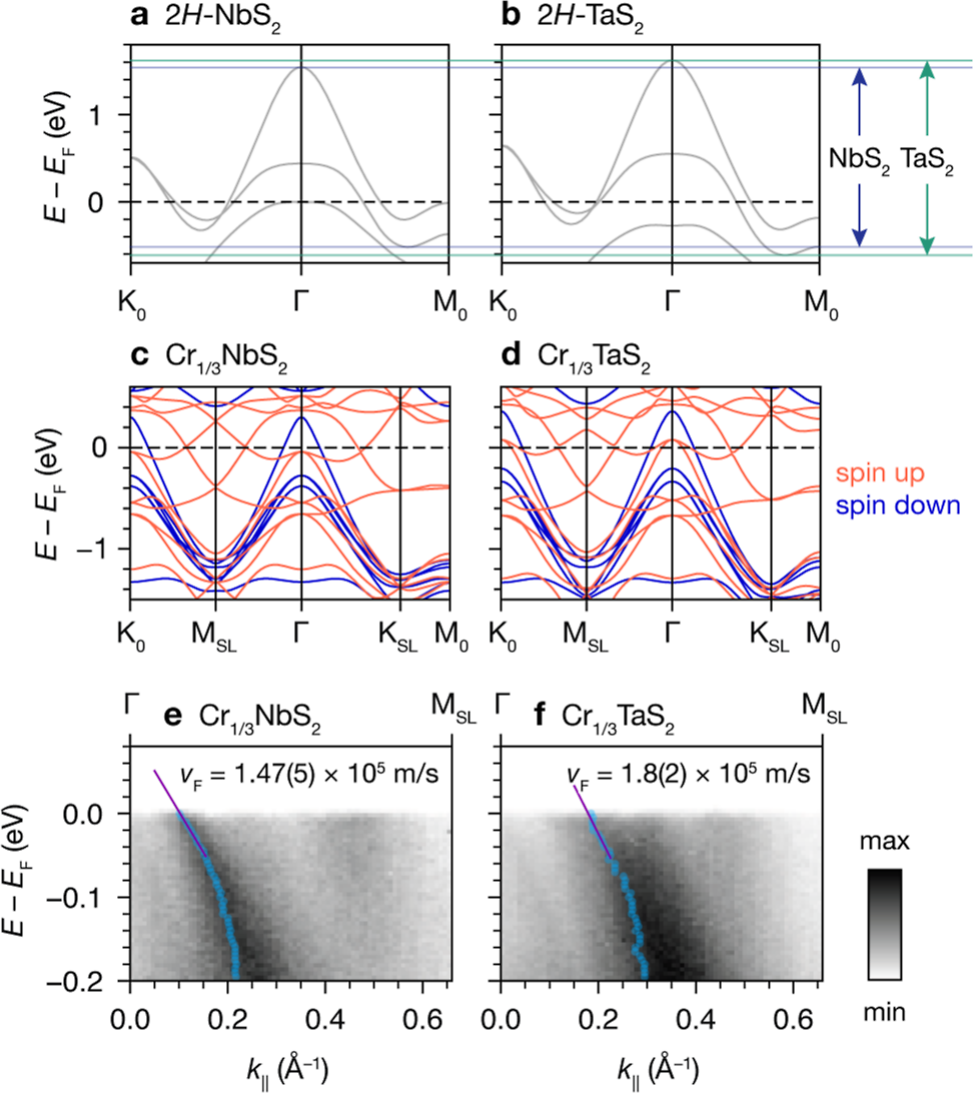
(a) and (b) DFT band structures of 2*H*-NbS_2_ and 2*H*-TaS_2_, with maxima and minima of the bands crossing *E*_F_ indicated by solid navy and green lines, respectively. (c) and (d) Spin-polarized band structures for Cr_1/3_NbS_2_ and Cr_1/3_TaS_2_ in the FM state, with spin-up and spin-down bands indicated in red and blue, respectively. (e,f) ARPES dispersions of Cr_1/3_NbS_2_ and Cr_1/3_TaS_2_ (18 K, *h*ν = 46 eV), with blue circles indicating the peak center positions of the most intense features from MDC analysis. The Fermi velocities, *v*_F_, are obtained from linear fits to the centers between 0 and −50 meV.

To understand the relative differences between the band structures of Cr_1/3_NbS_2_ and Cr_1/3_TaS_2_, we started by comparing the host lattice materials, 2*H*-NbS_2_ and 2*H*-TaS_2_. DFT band structure calculations of 2*H*-NbS_2_ and 2*H*-TaS_2_ show that the bands crossing *E*_F_ in 2*H*-TaS_2_ are more dispersive compared to the analogous bands in 2*H*-NbS_2_. This can be clearly visualized by comparing the relative spread of the maxima and minima of these respective bands, as illustrated in Figure 3a,b: the more dispersive bands in 2*H*-TaS_2_ have a higher-energy maximum and lower-energy minimum compared to 2*H*-NbS_2_. These bands have predominantly Nb or Ta  and *d*_*xy*_/ character, with additional contribution from S p states.^39,40^

Next, we calculated the band structures of the Cr-intercalated materials and compared the results to our ARPES data. Due to the surface-sensitive nature of ARPES, we do not expect to experimentally resolve signatures of the CHM state, that is, out-of-plane spin textures with length scales on the order of tens of nanometers. Hence, we use spin-polarized band structure calculations of Cr_1/3_NbS_2_ and Cr_1/3_TaS_2_ in their FM states, with the magnetization vector along [100], as proxies for the electronic structure near the surface (Figures 3c,d and S5 and S6). Three distinct changes are evident in the DFT band structures of Cr_1/3_NbS_2_ and Cr_1/3_TaS_2_ compared to the host lattices: (1) folding due to the  superlattice potential, (2) raising of *E*_F_ due to electron transfer from Cr to the host lattice, and (3) introduction of new bands crossing *E*_F_ due to Cr-derived states and FM exchange splitting.

Although the Cr-intercalated materials have more complex electronic structures than the host lattices, DFT calculations show that the Ta analogue again has more dispersive bands than the Nb analogue. The amount of charge transfer from Cr to the host lattice is very similar for both materials, as shown by Bader charge analysis (Tables S4 and S5), as well as the calculated and experimental magnetic moments (Tables S6 and S7). Thus, the shift of *E*_F_ upon intercalation is almost identical. This results in larger hole pockets around Γ and K_0_ in Cr_1/3_TaS_2_ than Cr_1/3_NbS_2_ and an extra spin-up band crossing *E*_F_ at Γ and K_0_ in Cr_1/3_TaS_2_. Notably, the ARPES dispersions of Cr_1/3_NbS_2_ and Cr_1/3_TaS_2_ at 18 K (*h*ν = 46 eV) show clearly that the most intense hole pocket around Γ in the Γ–M_SL_ direction is considerably larger at *E* = *E*_F_ in Cr_1/3_TaS_2_ compared to Cr_1/3_NbS_2_ (Figure 3e,f), with Fermi wavevectors, *k*_F_, of 0.10 Å for Cr_1/3_NbS_2_ and 0.19 Å for Cr_1/3_TaS_2_. By fitting the momentum distribution curves (MDCs) to Lorentzians between 0 and −50 meV, we extracted Fermi velocities, *v*_F_, of 1.47(5) × 10^5^ m/s for Cr_1/3_NbS_2_ and 1.8(2) × 10^5^ m/s for Cr_1/3_TaS_2_—thus experimentally quantifying the relative band dispersions between the two systems. The larger experimental *v*_F_ for the Ta analogue mirrors the relative trends from the DFT band structures.

### Orbital Character Assignments

**Figure 4:**
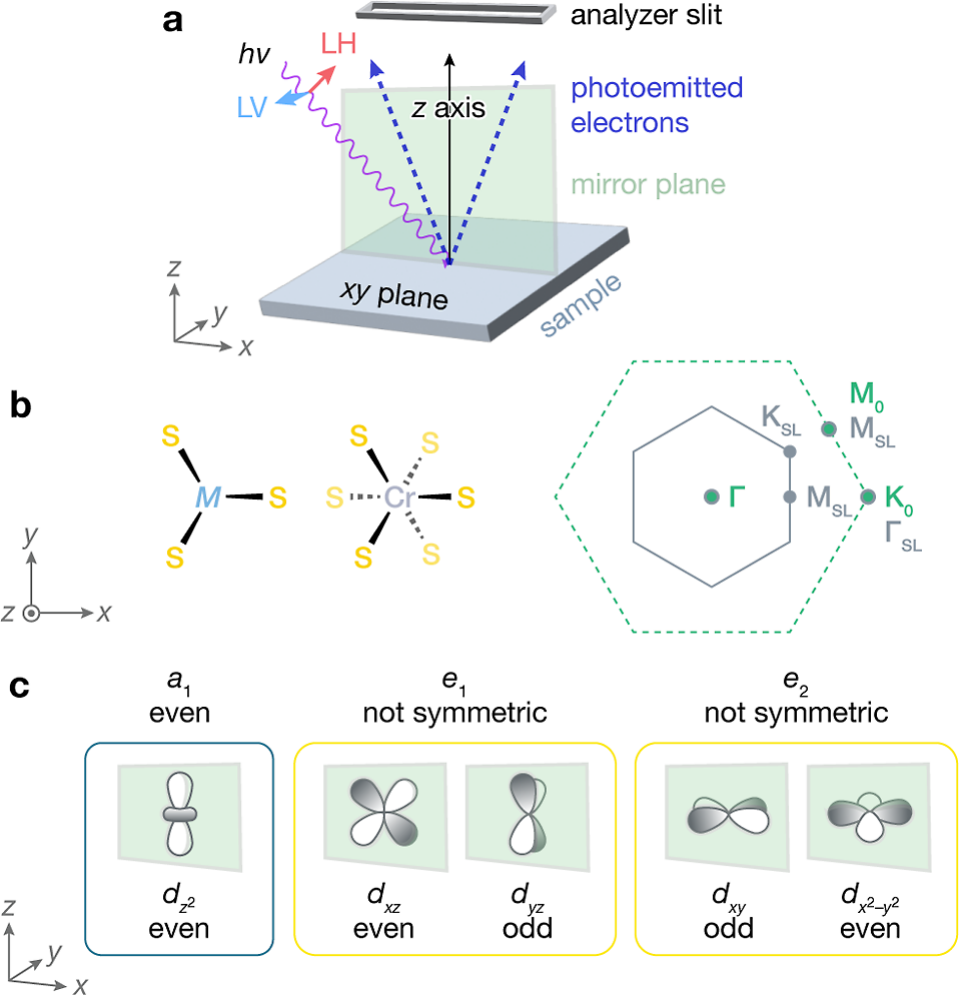
(a) Geometry of ARPES data collection for a horizontal analyzer slit aligned with the *xz* scattering plane of the sample. (b) Real space projections of local coordination environments for M = Nb or Ta and Cr, and surface Brillouin zones for the primitive lattice (dotted green) and  superlattice (solid gray). (c) Symmetries of d orbitals for M = Nb or Ta and Cr for the scattering plane defined as the *xz* plane aligned with the Γ–K_0_ direction.

To gain insight into the orbital character of the bands, we studied their polarization dependence in ARPES. For the photoemission process, the matrix element term can be described by
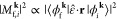
where ε̂ is the unit vector along the polarization direction of the light.^45^ The final state wavefunction of the photoelectron, ϕ_f_^**k**^, can be described by a plane-wave state, *e*^*i***kr**^, with even parity with respect to the mirror plane defined by the analyzer slit and the normal to the sample surface (Figure 4a). To obtain a nonvanishing matrix element, ε̂ must be even (odd) for an even (odd) initial state wavefunction, ϕ_*i*_^**k**^. Based on the symmetry operations of space group *P*6_3_22 (point group *D*_6_), and taking *z* to be parallel to the crystallographic *c*-axis, we expect the even *a*_1_ states of both Cr and Nb or Ta to be visible with linear horizontal (LH) polarized light (even ε̂) but not linear vertical (LV) polarized light (odd ε̂).

The *e*_1_ (*d*_*xz*_, *d*_*yz*_) and *e*_2_ (*d*_*xy*_, ) sets are not symmetric overall with respect to any scattering plane containing the sample surface normal. We illustrate this by considering a horizontal analyzer slit aligned to the Γ–K_0_ direction and defining *x* as parallel to the crystallographic *a*-axis of the superlattice unit cell. The resulting scattering plane is the *xz* plane (Figure 4a) and contains M–S and Cr–S bonds (Figure 4b). As shown in Figure 4c, the *d*_*xz*_ and  orbitals are even with respect to the *xz* plane, but the *d*_*yz*_ and *d*_*xy*_ orbitals (the other components of the *e*_1_ and *e*_2_ sets) are odd. Thus, the *e*_1_ and *e*_2_ sets are not symmetric collectively and may be visible with both LH and LV polarization.

**Figure 5:**
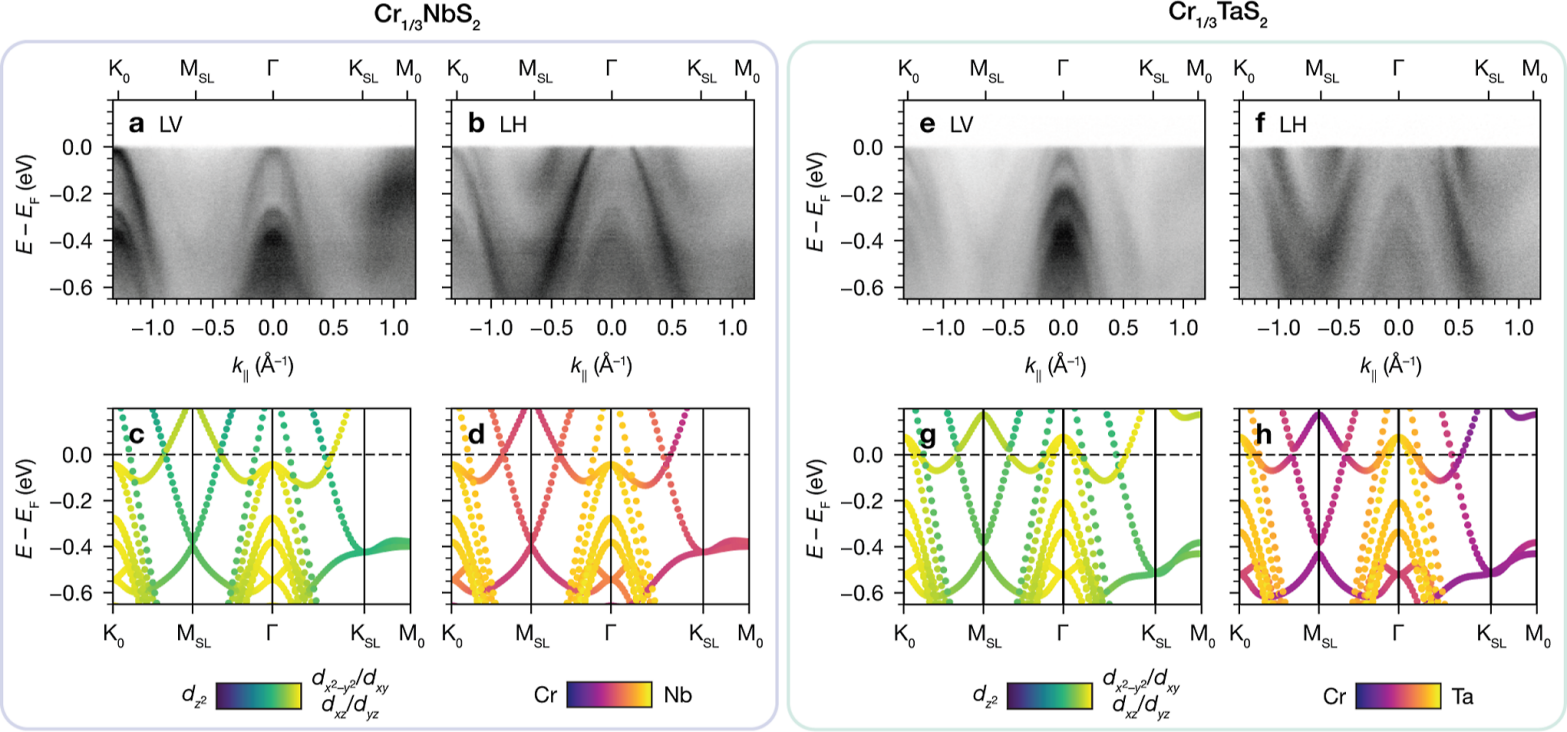
(a) and (b) ARPES band dispersions for Cr_1/3_NbS_2_ with LV and LH polarized photons, respectively (18 K, *h*ν = 79 eV). (c) and (d) DFT orbital-projected band structures of Cr_1/3_NbS_2_ in the FM state, showing in-plane vs out-of-plane character, and Cr vs Nb character, respectively. (e–h) The same as (a–d) for Cr_1/3_TaS_2_, and Cr vs Ta character in (h).

ARPES data of Cr_1/3_NbS_2_ measured with LV polarization (Figure 5a) show stronger intensity from the innermost parabolic bands centered at Γ and especially K_0_. In contrast, with LH polarization (Figure 5b), the sharp outermost dispersive bands around Γ and K_0_ are more prominent, as well as two sets of more diffuse electron pockets with minima at M_SL_ and K_SL_. To compare with the polarization-dependent ARPES data, we plotted the orbital-projected DFT band structure as a function of even  vs not symmetric (*d*_*xy*_/ and *d*_*xz*_/*d*_*yz*_) states in Figure 5c. At Γ/K_0_, the innermost parabolic bands have predominantly *d*_*xy*_/ and *d*_*xz*_/*d*_*yz*_ character, whereas the outermost dispersive bands and electron pockets have more  character. These parities are qualitatively consistent with the experimentally observed polarization dependence. The DFT band structure as a function of Cr vs Nb character (5d) indicates that all of the parabolic bands at Γ/K_0_ are predominantly Nb-derived, while the electron pockets with minima at M_SL_ and K_SL_ are composed of mixed Cr and Nb states. The polarization dependence of the host lattice bands more visible in LV polarization is consistent with *d*_*xy*_/ states folded to Γ from K_0_ by the  superlattice potential.^39^

The polarization-dependent ARPES data for Cr_1/3_TaS_2_ are similar to those for Cr_1/3_NbS_2_. The innermost bands at Γ and K_0_ are more prominent in LV polarization (Figure 5e), whereas the outer bands around Γ and K_0_ and more diffuse electron pockets with minima at M_SL_ and K_SL_ are more intense in LH polarization (Figure 5f). The orbital-projected DFT band structure reveals analogous parities to the Nb analogue (Figure 5g) and similar atomic parentage (Figure 5h), albeit with more Cr character in the vicinity of *E*_F_. We note that significant Cr contributions and Cr–Nb or Cr–Ta hybridization are observed in both materials in the energy range of interest.

**Figure 6:**
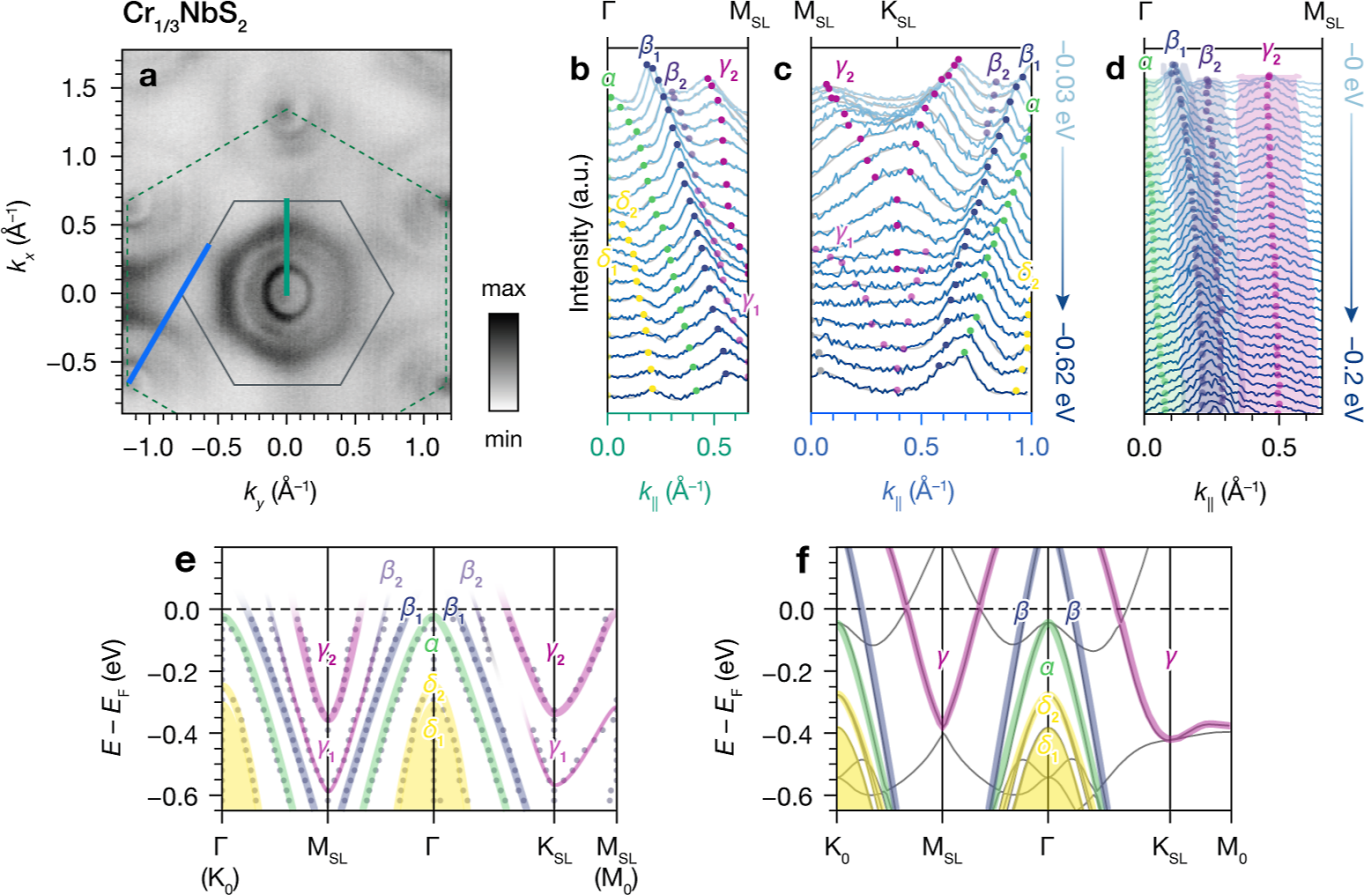
(a) ARPES Fermi surface of Cr_1/3_NbS_2_. Dashed and solid overlaid lines indicate the primitive and  superlattice Brillouin zones, respectively. (b,c) MDCs along the Γ–M_SL_ and M_SL_–K_SL_ directions (cuts indicated by bold blue and teal lines in (a)). Gray lines are multi-Lorentzian fits, and colored circles indicate peak center positions, with band assignments labeled. (d) MDCs along the Γ–M_SL_ direction (18 K, *h*ν = 46 eV). Shaded regions correspond to the full width at half maximum values determined by multi-Lorentzian fits. (e) Sketch of the proposed band structure of Cr_1/3_NbS_2_ derived from the MDC fits (peak center positions shown by gray circles). (f) DFT band structure of Cr_1/3_NbS_2_, with corresponding band assignments from MDC analysis indicated by colored overlays.

For a more quantitative enumeration of the bands near *E*_F_ observed in ARPES, we fitted the MDCs of the Cr_1/3_NbS_2_ data collected with LH polarization using multiple Lorentzian peaks along the cuts shown in Figure 6a–c. We refer to the dispersive features around Γ near *E*_F_ as the α, β, and γ bands, respectively, and two parabolic bands below *E*_F_ as δ_1_ and δ_2_. Comparison of the full width at half maximum (FWHM) values from fits to the Γ–M_SL_ MDCs within 200 meV of *E*_F_ indicates that the two middle bands have similar FWHMs, while the outermost band (corresponding to the shallow electron pocket) is considerably broader (Figure 6d). We therefore assign the middle two features as split β_1_ and β_2_ bands in the vicinity of *E*_F_. At higher binding energies, the MDCs can be fitted well with two copies of the electron pocket bands split by about 250 meV, which we refer to as γ_1_ and γ_2_. Comparing the peak center positions from MDC fitting (Figure 6e) with the DFT band structure (Figure 6f) shows good qualitative agreement, other than the apparent doubling of the β and γ bands observed in the ARPES data.

### Temperature Evolution of the Band Structure

**Figure 7:**
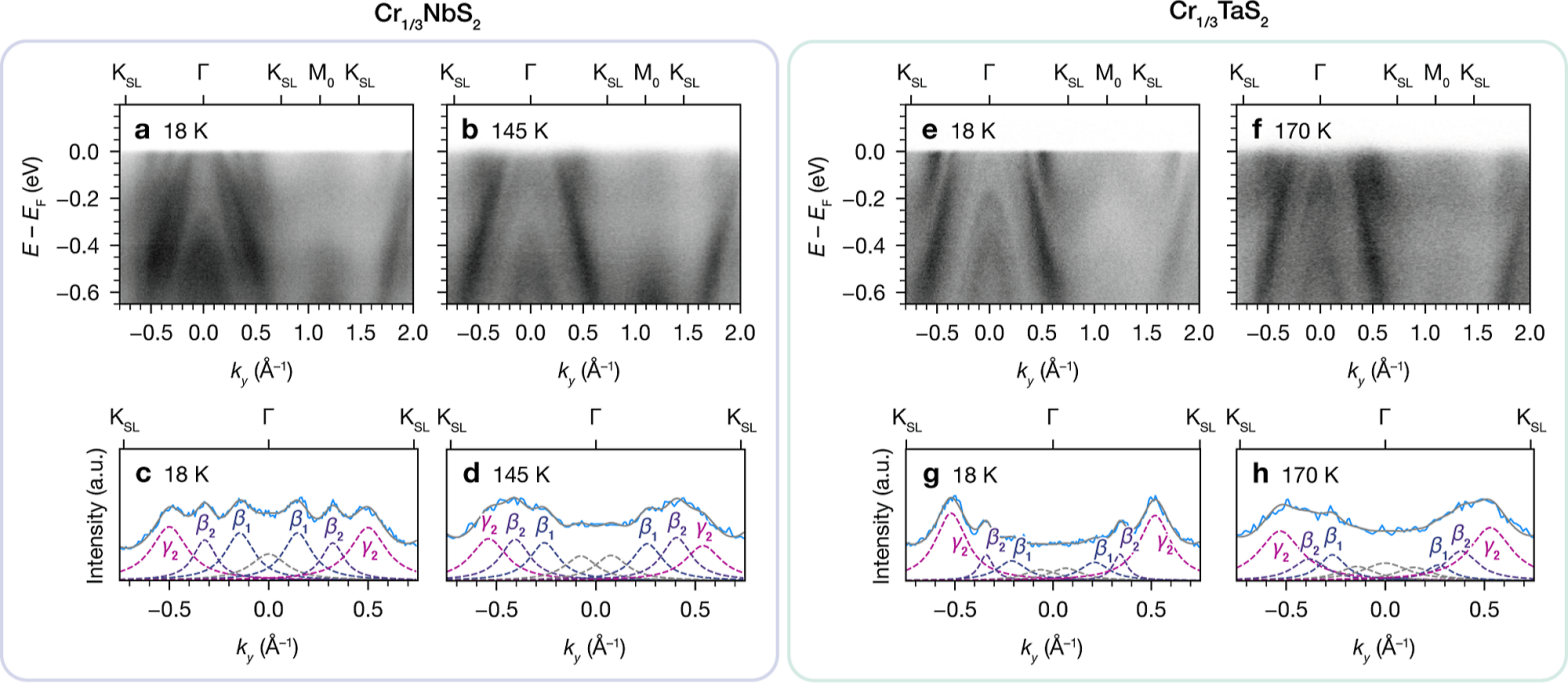
(a,b) ARPES dispersions for Cr_1/3_NbS_2_ taken at 18 and 145 K, respectively. (c,d) MDCs for Cr_1/3_NbS_2_ for *E* – *E*_F_ = −15 meV, and fits to multiple Lorentzian peaks (dotted lines), taken at 18 and 145 K, respectively. (e,f) ARPES dispersions for Cr_1/3_TaS_2_ taken at 18 and 170 K, respectively. (g,h) MDCs for Cr_1/3_TaS_2_ for *E* – *E*_F_ = −15 meV, and fits to multiple Lorentzian peaks (dotted lines), taken at 18 and 170 K, respectively. All data were measured with *h*ν = 79 eV and LH polarization.

Due to the aforementioned band splitting, we sought to probe the effect of magnetic ordering on the electronic structures of Cr_1/3_NbS_2_ and Cr_1/3_TaS_2_ by comparing ARPES data collected below and above *T*_C_. The dispersion of Cr_1/3_NbS_2_ at 18 K (Figure 7a) vs 145 K (Figure 7b) shows that the hole pockets around Γ appear smaller at 18 K compared to 145 K. Nevertheless, multi-Lorentzian fits to the MDCs at *E* – *E*_F_ = −15 meV show that the outer dispersive bands around Γ crossing *E*_F_ display the same splitting at 18 and 145 K, as indicated by the labeled β_1_, β_2_, and γ_2_ peaks in Figure 7c,d. A similar change in hole pocket sizes is evident in the dispersions of Cr_1/3_TaS_2_ at 18 K (Figure 7e) and 170 K (Figure 7f). As with Cr_1/3_NbS_2_, fitting the MDCs at *E* – *E*_F_ = −15 meV indicates that the splitting of the outer bands is observed at both 18 and 170 K (Figure 7g,h). For all the MDCs, we modeled the inner features around Γ with Lorentzian peaks as well, but we note that ascertaining the effects of temperature on these bands is more challenging due to the lower intensities and a non-negligible background component from inelastic scattering. Nonetheless, the persistence of the β_1_, β_2_, and γ_2_ splitting above *T*_C_ and the consistency in its magnitude for both materials prompted us to consider non-magnetic origins.

### ARPES Measurements with Micron-Scale Probes

**Figure 8:**
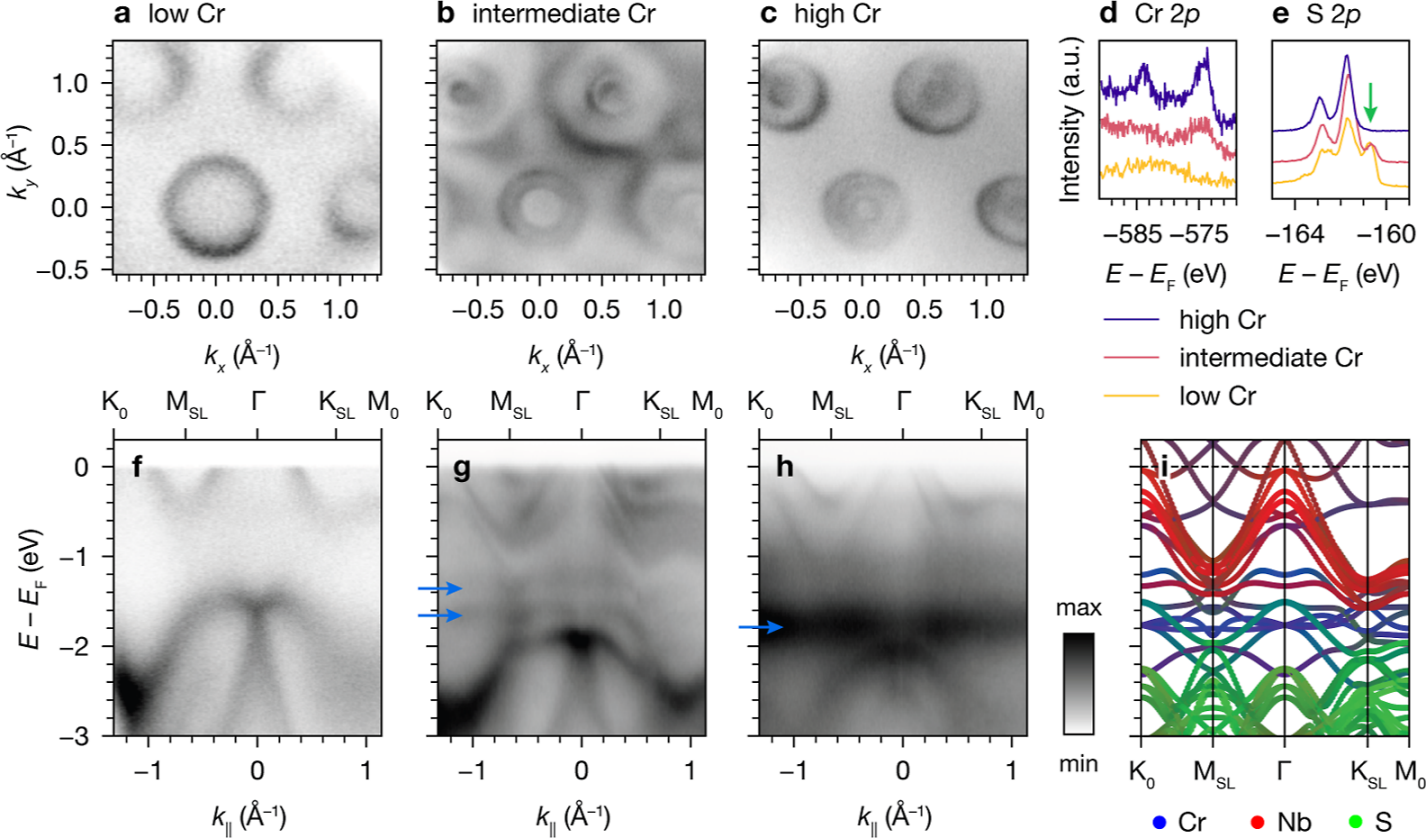
(a–c) ARPES Fermi surfaces of Cr_1/3_NbS_2_ taken in regions of low, intermediate, and high surface Cr coverage, respectively (20 K, *h*ν = 120 eV). (d,e) Cr 2p and S 2p core-level spectra, respectively, with the green arrow in (e) indicating the S 2p feature at low binding energy. (f–h) ARPES band dispersions for the same regions with low, intermediate, and high surface Cr coverage shown in (a–c). Blue arrows in (g) and (h) indicate non-dispersive features. (i) DFT orbital-projected band structure for Cr_1/3_NbS_2_ showing atomic origin of bands.

Motivated by the polar nature of these materials and the observation of unexplained band splitting, we carried out microARPES experiments on Cr_1/3_NbS_2_ with a smaller beam size (2–15 μm) to investigate the possible impact of nonuniform sample surfaces. We identified three types of distinct areas based on their Fermi surfaces (Figure 8a–c), core-level spectra (Figure 8d,e), and band dispersions (Figure 8f–h). Spots with the simplest Fermi surfaces and the largest hole pockets around Γ and K_0_ (Figure 8a) have the weakest Cr 2p core level spectra (Figure 8d). Spots with Fermi surfaces representative of the majority of the samples, with the aforementioned β and γ band doubling (Figure 8b), exhibit Cr 2p core-level signals of intermediate intensity. Finally, spots with Fermi surfaces missing the broadest outermost pockets around Γ and K_0_ (Figure 8c) show the strongest Cr 2p core level spectra. Based on the Cr core level intensities, these areas appear to correspond to low, intermediate, and high relative Cr surface concentrations, respectively. The trend in the S 2p core levels from the same spots corroborates this assignment: with decreasing Cr surface coverage, an S peak at lower binding energies grows (indicated by the green arrow in Figure 8e), consistent with more reduced S sites on the surface that are not sharing electron density with Cr.

The ARPES dispersions from these spots also exhibit notable differences. The “low Cr” spot (Figure 8f) exhibits less  superlattice reconstruction than the other spots (as seen from the apparent threefold symmetry around K_0_ and different-sized hole pockets at Γ and K_0_) and resembles 2*H*-NbS_2_ with *E*_F_ shifted up by approximately 250 meV.^39^ The intense “X”-shaped feature at Γ located at about −1.6 eV in “low Cr” is shifted down to about −2.0 eV in both “intermediate Cr” (Figure 8g) and “high Cr” (Figure 8h) consistent with the latter two samplings more electron-doped states on average. In the “intermediate Cr” spot, the γ band electron pockets near *E*_F_ are split by about 250 meV as they are in other spectra measured with larger beam sizes. In the “high Cr” spot, the electron pockets near *E*_F_ are not noticeably split; instead, only a single set of features resembling the lower γ_1_ band in “intermediate Cr” is observed. Additionally, the presence of flat bands in the “intermediate Cr” and “high Cr” spots (where they are especially prominent), as indicated by the blue arrows in Figure 8g,h, coincides with Cr states in the orbital-projected DFT band structures (Figure 8i), lending further support to the surface coverage assignments.

## Discussion

### Band Structure and Magnetic Exchange Interactions

Taking the results from both ARPES and DFT into account, the most pronounced difference in the band structures of Cr_1/3_NbS_2_ and Cr_1/3_TaS_2_ is the more dispersive bands in the Ta analogue. The origin appears to be steeper dispersions in 2*H*-TaS_2_ compared to 2*H*-NbS_2_; i.e., the relative band dispersions of the host lattice materials are retained after Cr intercalation. This trend can be attributed to better overlap facilitated by more extended Ta 5d orbitals compared to the Nb 4d orbitals. For a more detailed explanation, we discuss the salient bonding interactions in both host lattice materials in brief.

**Figure 9:**
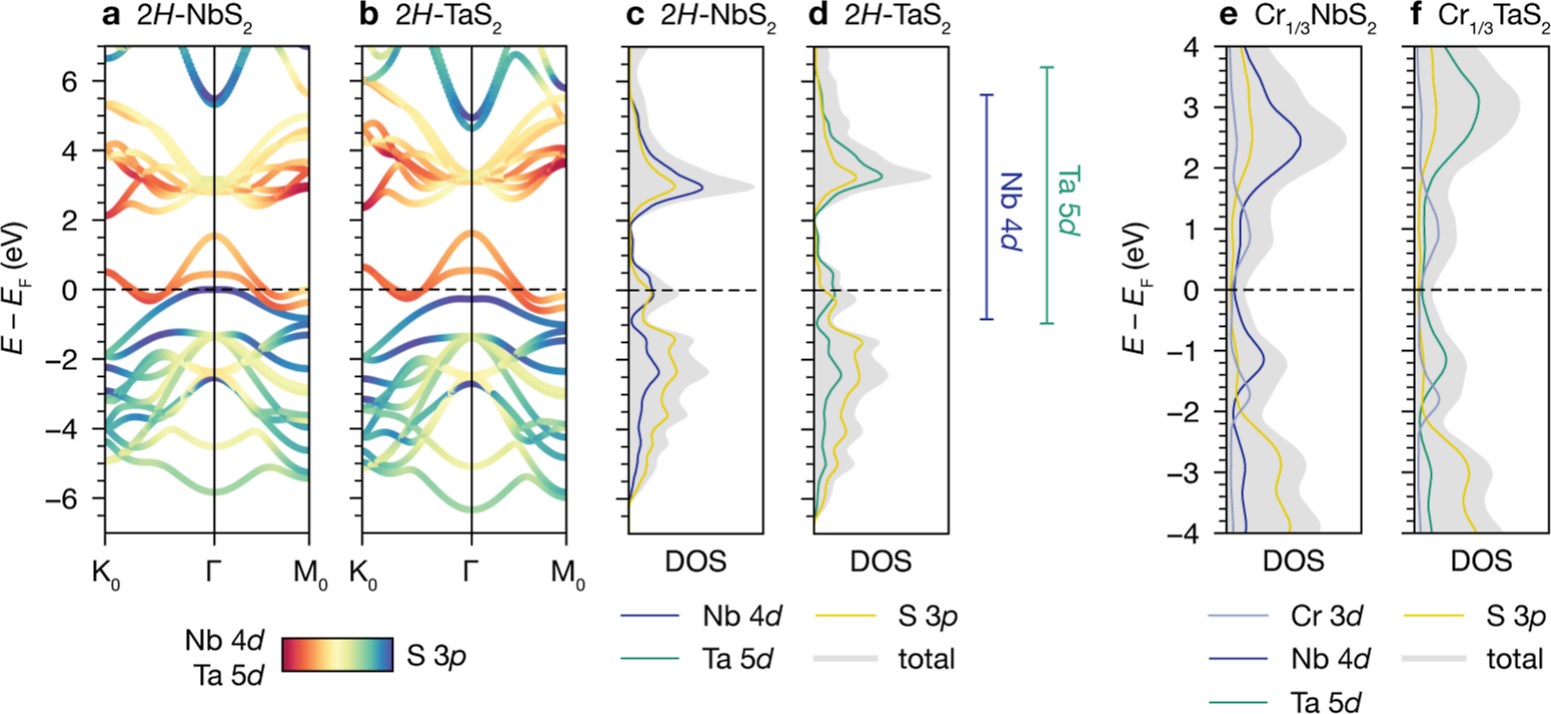
(a) Orbital-projected band structure calculation for 2*H*-NbS_2_, showing Nb 4d and S 3p characters. (b) Orbital-projected band structure calculation for 2*H*-TaS_2_, showing Ta 5d and S 3p character. (c) Projected DOS calculation for 2*H*-NbS_2_. (d) Projected DOS calculation for 2*H*-TaS_2_. The energy spread of the Nb 4d and Ta 5d bands is indicated on the right. (e,f) Projected DOS calculations for Cr_1/3_NbS_2_ and Cr_1/3_TaS_2_.

The bands within about 6 eV of the Fermi level in 2*H*-MS_2_ (M = Nb or Ta) are composed of M d states and S 3p states, indicating that M–S d–p and M–M d–d interactions are those relevant to determining the strength of the bonding and the resulting dispersivity of the bands. Mixing among the M d orbitals results in the formation of a hybridization gap within the d manifold: the bands crossing *E*_F_ are composed of , *d*_*xy*_, and  orbitals, while the higher-lying d bands have more *d*_*xz*_ and *d*_*yz*_ character.^31^ TaS_2_ has a slightly smaller in-plane lattice constant,^46,47^ and the 5d orbitals are more spatially extended than the 4d orbitals in NbS_2_. This leads to better relative overlap in the Ta analogue, both in terms of M–S d–p bonds and next-nearest-neighbor M–M d–d interactions. Hence, overall, the d manifold of TaS_2_ is more dispersive than that of NbS_2_, as shown in the band structure and density of states (DOS) calculations in Figure 9c,d. In turn, the bands crossing *E*_F_ are also more dispersive in the Ta analogue.

These arguments can also be used to explain why the host lattice-derived bands are more dispersive in Cr_1/3_TaS_2_ than in Cr_1/3_NbS_2_, which we have observed in ARPES and DFT (Figure 9e,f). From the crystal structures, the Ta–S bonds in Cr_1/3_TaS_2_ are slightly shorter (2.488(3) Å on average) than the Nb–S bonds in Cr_1/3_NbS_2_ (2.4931(11) Å on average), and the in-plane lattice constant in Cr_1/3_TaS_2_ is slightly smaller (5.7155(5) Å vs 5.7400(7) Å), suggestive of stronger Ta–S orbital overlap compared to Nb–S overlap. In addition, the in-plane electrical conductivity of Cr_1/3_TaS_2_ is more than an order of magnitude higher than that of Cr_1/3_NbS_2_, which is consistent with the more dispersive bands and larger hole pockets in the Ta analogue expected from this analysis.^18,20^

The more dispersive bands in Cr_1/3_TaS_2_ compared to Cr_1/3_NbS_2_ ultimately result in different Fermi wavevectors, *k*_F_. Nevertheless, the two materials have qualitatively analogous magnetic phase diagrams. This finding supports the notion that the RKKY interaction does not adequately describe exchange coupling in these materials.^18,25−27,29^ According to the RKKY formalism, the sign and magnitude of *J* would depend closely on the magnitude of *k*_F_, which is evidently not the case for Cr_1/3_NbS_2_ and Cr_1/3_TaS_2_. Furthermore, we find evidence of substantial Cr character in bands crossing *E*_F_. This result points to the salience of hybridization and orbital effects on magnetism in these materials.

To investigate the relative strengths of magnetic exchange interactions, we used a minimal model considering nearest neighbor and next-nearest neighbor interactions in- and out-of-plane (see the Supporting Information and Figures S8–S10 and Tables S8–S11 for computational details).^48^ We find that the total *J* is larger for the Ta analogue (−3.96 meV) than the Nb analogue (−3.72 meV), driven primarily by larger out-of-plane *J*_⊥_. This result directly demonstrates stronger FM coupling in Cr_1/3_TaS_2_ compared to Cr_1/3_NbS_2_, consistent with the higher *T*_C_ of Cr_1/3_TaS_2_.

The magnetic soliton wavelength and response of the spin textures to the magnetic field are ultimately determined by the ratio of *D*, the DM interaction term, to *J*_⊥_, the out-of-plane FM exchange constant.^34,35^ Although we find that *J*_⊥_ is larger in Cr_1/3_TaS_2_, the soliton wavelength is nevertheless shorter.^19,21,38^ This observation implies that the ratio *D*/*J*_⊥_ is considerably larger in the Ta analogue than in the Nb analogue; i.e., *D* increases more than *J*_⊥_ in going from Nb to Ta. Larger SOC in the Ta analogue is directly responsible for the larger *D*.^19−21^ Although we do not directly probe SOC in ARPES, it appears to affect the length and energy scales of the chiral spin textures more than changes in *J*.

It has been established that a delicate balance exists between disorder and vacancies in the Cr superlattice and the integrity of the desired spin textures.^22,38,49^ Instead, co-intercalation of another species into the interstitial space^50^ or substitutional doping on the TMD sublattice could constitute other pathways toward tuning SOC or the filling level while maintaining a well-ordered Cr superlattice (and hence a globally defined *D*). We note additionally that the sensitivity of the observed surface states on Cr concentration—as discussed in more detail in the next section—suggests that the surface electronic structure is amenable to tuning through further functionalization. Altogether, we conclude that SOC and hybridization are chiefly responsible for quantitative differences in the magnetic properties of Cr_1/3_NbS_2_ and Cr_1/3_TaS_2_. These factors may also be relevant to the disparate magnetism observed between the Nb and Ta analogues in other intercalated TMDs (e.g., Fe_1/3_NbS_2_ is an antiferromagnet, whereas Fe_1/3_TaS_2_ is a ferromagnet^12,51−53^).

### Exchange Splitting vs Surface Termination Effects

Previous ARPES studies on Cr_1/3_NbS_2_ have also reported band splitting near *E*_F_ that appears similar to our assignment of β_1_, β_2_, and γ_2_ bands. These works interpreted this phenomenon as exchange splitting.^25−27^ However, we found good agreement between the exchange splitting predicted by our FM spin-polarized DFT band structures and observed in our ARPES results. Specifically, according to the spin-polarized DFT band structure calculations shown in Figure 3c,d, the α and β bands (as labeled in Figure 6f) are an exchange–split pair. Because of different mixing in the spin-up and spin-down channels, α has more *d*_*xy*_/ character, while β has more  character (Figure 5c,g). The corresponding bands observed in ARPES show the expected polarization dependence: the α band, which just touches *E*_F_, is much more visible in LV polarization (Figure 5a,e), whereas the β band is more prominent in LH polarization (Figure 5b,f). We also calculated the band structures with SOC, which did not explain the additional bands (Figure S11). This prompted us to consider alternative explanations for the observation of more bands in ARPES than predicted by DFT.

**Figure 10:**
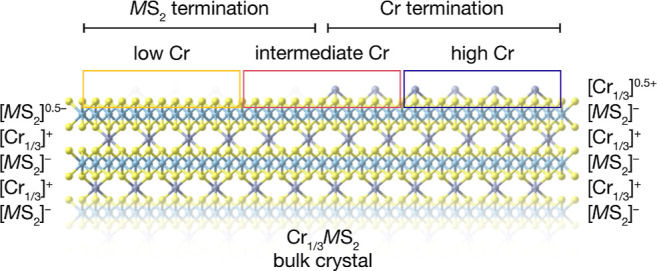
Schematic illustration of local Cr clustering on cleaved surfaces of Cr_1/3_MS_2_ samples, with two distinct regions corresponding to predominantly MS_2_ and Cr terminations. Formal charges given for the two regions are based on a simple electron-counting picture, assuming completely full (absent) Cr coverage in the Cr- (MS_2_-) terminated regions. The “low Cr,” “intermediate Cr,” and “high Cr” sampling areas (colored boxes) thus have average surface stoichiometries of 0, 1/6, and 1/3.

Instead, taking the polar nature of intercalated TMDs into account, we surmised that the observed doubling of β_1_/β_2_ and γ_1_/γ_2_ bands could be attributed to surface termination effects. Previous work suggests that spatially distinct areas of Cr- and MS_2_-termination exist on cleaved surfaces: STM studies have observed islands of intercalants on cleaved crystals of intercalated TMDs,^25,54^ and recent ARPES studies of V_1/3_NbS_2_^44^ and Co_1/3_NbS_2_^55^ reported termination-dependent surface states. Thus, to understand the expected effects of surface termination on the ARPES data, we consider the charge distributions for Cr-terminated and MS_2_-terminated surfaces. As summarized in Figure 1a, the Cr^3+^ centers formally donate one electron per MS_2_ formula unit, leading to alternating layers with +1 and −1 formal charges. The phenomenon of charge redistribution at polar-to-nonpolar interfaces to prevent a polar catastrophe (i.e., diverging electrostatic potential) is well-documented,^56^ and we expect analogous redistribution to occur at both the [MS_2_]–vacuum interface and the [Cr_1/3_]–vacuum interface. Assuming fully occupied (unoccupied) Cr sites for the Cr- (MS_2_-) terminated regions, the surface formal charges expected from simple electron counting are shown in Figure 10. In short, MS_2_-terminated regions should exhibit MS_2_-derived surface states that are hole-doped relative to the bulk, originating from partial electron transfer from the surface TMD layer to compensate for its polarity.

Building upon this picture, we propose that the three distinct spectral signatures observed with mesoscopic probes (Figure 8) can be explained by sampling two different surface terminations, as illustrated in Figure 10. The “low Cr” and “high Cr” spots correspond to areas with almost exclusively MS_2_ termination and Cr termination, respectively. The “intermediate Cr” spots contain both terminations, resulting in doubled β and γ bands that are also observed in data collected with larger probes. The β and γ bands at lower binding energies are associated with MS_2_-terminated regions. Such effects are broadly consistent with those observed on surfaces of other polar layered materials.^57^ Hence, we propose that these additional band doublings do not reflect magnetic ordering but rather originate from charge redistribution. More generally, the possible contribution of surface states should be considered in other cases where unexpected bands are observed in ARPES studies on magnetic intercalated TMDs.

## Conclusions

The electronic structures of the chiral helimagnets Cr_1/3_NbS_2_ and Cr_1/3_TaS_2_ have been investigated using ARPES and DFT. Compared to the host lattice materials 2*H*-NbS_2_ and 2*H*-TaS_2_, the Cr-intercalated materials exhibit band folding from the  Cr superlattice, higher *E*_F_ from electron transfer from Cr to the host TMD, exchange splitting from the in-plane FM ordering of Cr moments, and new bands from Cr-derived states. The chief difference between the band structures of 2*H*-NbS_2_ and 2*H*-TaS_2_—more dispersive bands in the Ta analogue—is retained after Cr intercalation, resulting in a higher *v*_F_ and a larger *k*_F_ in Cr_1/3_TaS_2_. The fact that the magnetic phase diagrams of the Nb and Ta materials are analogous despite these differences indicates that the RKKY interaction is not chiefly responsible for the magnetic exchange.

By studying the polarization dependence in ARPES and fitting the MDCs, we find that the experimentally observed band structures agree well with the orbital-projected DFT band structures. The primary features at *E*_F_ in both materials consist of dispersive hole pockets at Γ (and K_0_) and shallow electron pockets centered around K_SL_. Notably, many bands near *E*_F_ have significant Cr character in both materials, indicating that a rigid band model is insufficient for modeling the effects of Cr intercalation. Additional copies of bands crossing *E*_F_ that are not predicted by DFT are assigned to surface states originating from TMD-terminated regions. The observation of three distinct regions in ARPES experiments with smaller spot sizes is consistent with Cr, TMD, and mixed-surface terminations. These results indicate that the polar nature of the surfaces of intercalated TMDs affects the band splitting observed in ARPES data.

It has been well established that Cr_1/3_NbS_2_ and Cr_1/3_TaS_2_ have analogous magnetic phase diagrams with different energy scales and different wavelengths of magnetic solitons. Our results indicate that despite stronger FM coupling in Cr_1/3_TaS_2_, the larger DM interaction term—driven by the heavier host lattice and larger SOC, which should scale approximately with *Z*^4^—is chiefly responsible for establishing the quantitative differences in their magnetic properties. Nevertheless, further tuning of the magnetic exchange interactions and SOC via substitutional doping on the TMD sites or co-intercalation of other species may afford further control over their spin textures. Overall, the chiral helimagnets Cr_1/3_NbS_2_ and Cr_1/3_TaS_2_ are promising platforms for studying the interplay between electronic structure and the microscopic mechanisms underlying noncollinear magnetism.

## Methods

### Crystal Growth

Single crystals of Cr_1/3_NbS_2_ and Cr_1/3_TaS_2_ were grown using chemical vapor transport using iodine as a transport agent. For Cr_1/3_NbS_2_, high-purity powders of elemental Cr, Nb, and S in a 0.6:1:2 ratio and 5 mg/cm^3^ of I_2_ were sealed under vacuum in a fused quartz ampoule approximately 48 cm long. The ampoule was placed in a three-zone tube furnace with the hot end zone and middle zone maintained at 1050 °C and the cold (growth) zone maintained at 850 °C for 14 days before cooling to room temperature. For Cr_1/3_TaS_2_, high-purity powders of elemental Cr, Ta, and S in a 0.47:1:2.1 ratio and 2 mg/cm^3^ of I_2_ were sealed under vacuum in a fused quartz ampoule approximately 25 cm long. The ampoule was placed in a two-zone tube furnace with the hot zone maintained at 1100 °C and the cold (growth) zone maintained at 1000 °C for 14 days before cooling to room temperature. Shiny plate-shaped crystals with a silvery metallic luster and hexagonal habit up to 4 × 4 × 0.5 mm in size were obtained.

### Structural and Compositional Characterization

Single-crystal X-ray diffraction was collected on a Rigaku XtaLAB P200 with Mo Kα radiation at 295 K. Data reduction and scaling and empirical absorption correction were performed in CrysAlis Pro. Structures were solved by direct methods using SHELXT^58^ and refined against *F*^2^ on all data by full-matrix least squares with SHELXL.^59^ Raman spectroscopy was collected on a Horiba LabRAM HR Evolution with an ultra-low-frequency filter using 633 nm laser excitation and powers between 1 and 8 mW. Energy-dispersive X-ray spectroscopy was acquired on an FEI Quanta 3D FEG or a Scios 2 DualBeam scanning electron microscope with an accelerating voltage of 20 kV.

### Magnetometry

DC magnetization measurements were carried out on a Quantum Design Physical Property Measurement System Dynacool equipped with a 12 T magnet using either the Vibrating Sample Magnetometer option or the AC Measurement System II option. Single crystals were affixed to quartz sample holders with GE Varnish such that the magnetic field was applied perpendicular to the crystallographic *c* axis.

### ARPES Measurements

ARPES data were collected at the Quantum Materials Spectroscopy Centre (QMSC) of the Canadian Light Source (CLS) and at Beamline 7.0.2 of the Advanced Light Source (ALS) on both the microARPES and nanoARPES endstations using Scienta Omicron R4000 hemispherical electron analyzers. The beam spot sizes were approximately 20 × 100 μm at QMSC, 15 × 15 μm on microARPES, and 2 × 2 μm on nanoARPES. Results were reproduced on multiple samples at both beamlines with the exception of spatial variation observed with smaller spot sizes. Samples were cooled down to the base temperature of 20 K or below and cleaved in situ by carefully knocking off alumina posts affixed to the top surface of the sample with silver epoxy. All measurements were conducted at pressures lower than 5 × 10^–11^ Torr. The primary datasets were collected at photon energies of 46, 79, and 120 eV with LH and LV polarizations. Data analysis was performed using the PyARPES software package.^60^

### DFT Calculations

First-principles calculations were performed by using the open-source plane-wave code Quantum Espresso.^61^ The optimized norm-conserving Vanderbilt pseudopotentials from the PseudoDojo project^62,63^ were applied. The kinetic energy cut-off for wavefunctions was set to 86 Ry for all the self-consistent calculations; for these calculations, the experimental lattice constants obtained from X-ray diffraction were used.^46,47^ van der Waals correction was applied to account for the long-range interaction between layers.^64,65^ A Γ-centered 4 × 4 × 2 *k*-mesh was sampled in the Brillouin zone for both Cr_1/3_NbS_2_ and Cr_1/3_TaS_2_, and a 8 × 8 × 2 *k*-mesh for both 2*H*-NbS_2_ and 2*H*-TaS_2_. The Perdew–Burke–Ernzerhof functional^66^ of the spin-polarized generalized gradient approximation was used to describe the exchange correlation of electrons.

## Supporting Information Available

Crystallographic data from single crystal X-ray diffraction; Raman spectra; energy-dispersive X-ray spectroscopy data; *k*_z_-projected band structures; Bader charge analysis; calculated and experimental magnetic moments; exchange interaction analysis; band structures with spin–orbit coupling for Cr_1/3_NbS_2_ and Cr_1/3_TaS_2_ (PDF)
