# Supplementary material for: Comparative Electronic Structures of the Chiral Helimagnets Cr1/3NbS2 and Cr1/3TaS2
Source: Chem Mater. 2023 Aug 16;35(17):7239–51. doi: 10.1021/acs.chemmater.3c01564 (PMC10500995; doi:10.1021/acs.chemmater.3c01564)
Supplement: Supplementary file 1 — cm3c01564_si_001.pdf [file cm3c01564_si_001.pdf]

Supporting Information for

Comparative Electronic Structures of the Chiral Helimagnets $Cr_{1/3}NbS_2$ and $Cr_{1/3}TaS_2$

Lilia S. Xie[1,†], Oscar Gonzalez[1,†], Kejun Li[2], Matteo Michiardi[3,4], Sergey Gorovikov[5], Sae Hee Ryu[6], Shannon S. Fender[1], Marta Zonno[5], Na Hyun Jo[6,7], Sergey Zhdanovich[3,4], Chris Jozwiak[6], Aaron Bostwick[6], Samra Husremović[1], Matthew P. Erodici[1], Cameron Mollazadeh[1], Andrea Damascelli[3,4], Eli Rotenberg[6], Yuan Ping[8,2], and D. Kwabena Bediako[1,9,*]

[1] *Department of Chemistry, University of California, Berkeley, CA 94720, USA*

[2] *Department of Physics, University of California, Santa Cruz, CA, 95064, USA*

[3] *Quantum Matter Institute, University of British Columbia, Vancouver, BC V6T 1Z4, Canada*

[4] *Department of Physics and Astronomy, University of British Columbia, Vancouver, BC V6T 1Z1, Canada*

[5] *Canadian Light Source, Inc., 44 Innovation Boulevard, Saskatoon SK S7N 2V3, Canada*

[6] *Advanced Light Source, Lawrence Berkeley National Laboratory, Berkeley, CA 94720, USA*

[7] *Department of Physics, University of Michigan, Ann Arbor, Michigan 48109, USA*

[8] *Department of Materials Science and Engineering, University of Wisconsin, Madison, WI, 53706, USA*

[9] *Chemical Sciences Division, Lawrence Berkeley National Laboratory, Berkeley, CA 94720, USA*

[*] *Correspondence to: bediako@berkeley.edu*

[†] *These authors contributed equally to this work*

Contents

1 Single Crystal X-ray Diffraction **S3**

2 Raman Spectroscopy **S5**

3 Energy Dispersive X-ray Spectroscopy **S6**

4 Density Functional Theory Calculations **S7**

4.1 Bader Charge Analysis S10

4.2 Magnetic Moment S12

4.3 Exchange Interaction Analysis S13

4.4 Effect of Spin–Orbit Coupling on Band Structure S18

1 Single Crystal X-ray Diffraction

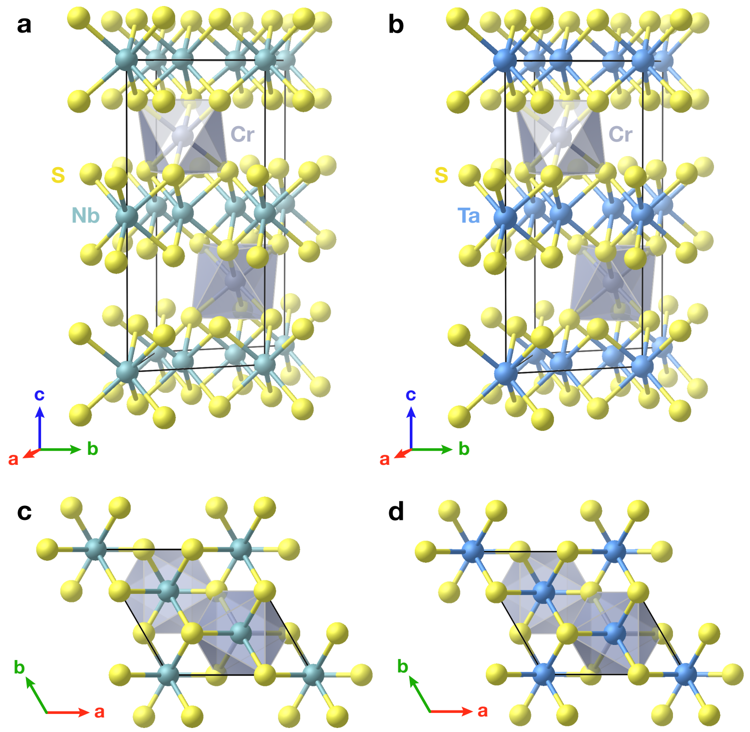

Figure S1: Crystal structures of $Cr_{1/3}NbS_2$ and $Cr_{1/3}TaS_2$ from single crystal X-ray diffraction. Representations are shown for $Cr_{1/3}NbS_2$ and $Cr_{1/3}TaS_2$, respectively, along the a crystallographic axis in (a) and (b), and c crystallographic axis in (c) and (d).

Table S1: Crystal data and structure refinement for $Cr_{1/3}NbS_2$ and $Cr_{1/3}TaS_2$.

	$Cr_{1/3}NbS_2$	$Cr_{1/3}TaS_2$
Empirical formula	$CrNb_3S_6$	$CrTa_3S_6$
Formula weight (g/mol)	523.09	787.21
Temperature (K)	293(2)	293(2)
Wavelength (Å)	0.71073	0.71073
Crystal system	Hexagonal	Hexagonal
Space group	$P6_322$	$P6_322$
a (Å)	5.7400(7)	5.7155(5)
c (Å)	12.1082(14)	12.1751(12)
Volume (Å^{-3})	345.49(9)	344.44(7)
Z	2	2
Density (calculated) (g/cm^3)	3.402	5.680
Absorption coefficient (mm^{-1})	8.082	50.733
$F(000)$	486	678
Crystal size (mm^3)	$0.033 \times 0.017 \times 0.013$	$0.119 \times 0.067 \times 0.025$
θ (°)	3.365 to 29.272	4.117 to 29.531
Index ranges	$-6 \leq h \leq 7$	$-7 \leq h \leq 7$
	$-7 \leq k \leq 6$	$-7 \leq k \leq 7$
	$-14 \leq l \leq 16$	$-16 \leq l \leq 16$
Reflections collected	2684	2641
Independent reflections	297	302
Completeness to $\theta_{\rm full}$	1.000	0.994
Absorption correction	Semi-empirical from equivalents	Semi-empirical from equivalents
Refinement method	Full-matrix least-squares on F^2	Full-matrix least-squares on F^2
Data / restraints / parameters	297 / 0 / 17	302 / 0 / 17
Goodness-of-fit on F^2	1.312	1.156
Final R indices [$I > 2\sigma(I)$]	$R_1 = 0.0331$, $wR_2 = 0.0848$	$R_1 = 0.0381$, $wR_2 = 0.1166$
R indices (all data)	$R_1 = 0.0435$, $wR_2 = 0.0880$	$R_1 = 0.0437$, $wR_2 = 0.1213$
Largest diff. peak and hole (e Å^{-3})	1.81 and −0.65	4.83 and −1.89

Table S2: Atomic coordinates, Wyckoff positions, and equivalent isotropic displacement parameters for $Cr_{1/3}NbS_2$.

Atom Labels	x	y	z	Site	$U_{\rm iso}$
Cr01	2/3	1/3	3/4	$2c$	0.0067(6)
Nb02	0	0	1/2	$2a$	0.0037(4)
Nb03	1/3	2/3	0.50283(6)	$4f$	0.00236(3)
S04	0.6680(3)	0.6675(3)	0.63086(11)	$12i$	0.0048(4)

Table S3: Atomic coordinates, Wyckoff positions, and equivalent isotropic displacement parameters for $Cr_{1/3}TaS_2$.

Atom Labels	x	y	z	Site	$U_{\rm iso}$
Cr01	2/3	1/3	3/4	$2c$	0.0098(7)
Ta02	0	0	1/2	$2a$	0.0047(5)
Ta03	1/3	2/3	0.50232(4)	$4f$	0.0048(5)
S04	0.6679(3)	0.6685(3)	0.6304(2)	$12i$	0.0058(7)

2 Raman Spectroscopy

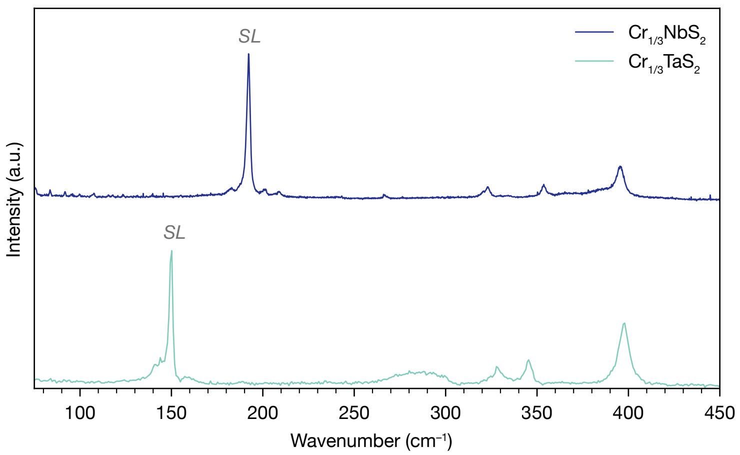

Figure S2: Raman spectra of $Cr_{1/3}NbS_2$ and $Cr_{1/3}TaS_2$, with modes associated with the $\sqrt{3} \times \sqrt{3}$ superlattice labeled as "SL."[1]

3 Energy Dispersive X-ray Spectroscopy

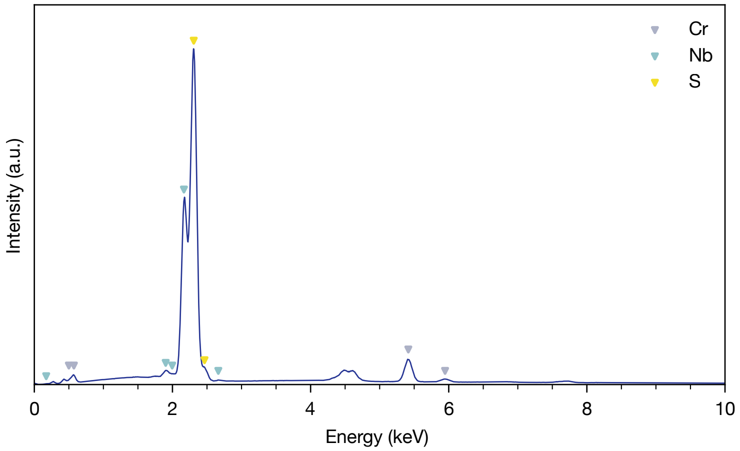

Figure S3: Representative dispersive X-ray spectroscopy data for a single crystal of $Cr_{1/3}NbS_2$ with peaks corresponding to Cr, Nb, and S labeled. The atomic ratio determined by fitting the Cr Kα_1, Nb Lα_1, and S Kα_1 peaks was 1.00:3.00:6.30., corresponding to a formula of $Cr_{0.33}NbS_{2.10}$.

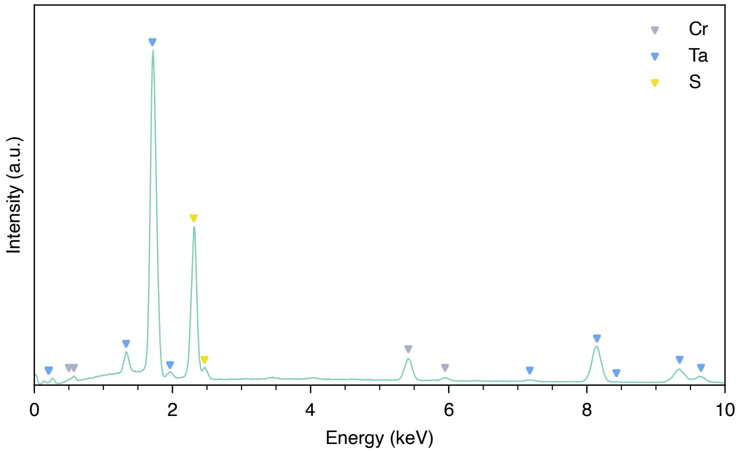

Figure S4: Representative dispersive X-ray spectroscopy data for a single crystal of $Cr_{1/3}TaS_2$ with peaks corresponding to Cr, Ta, and S labeled. The atomic ratio determined by fitting the Cr Kα_1, Ta Mα_1, and S Kα_1 peaks was 1.00:3.07:5.70, corresponding to a formula of $Cr_{0.33}TaS_{1.86}$.

4 Density Functional Theory Calculations

To understand the band structures obtained from ARPES measurements, first-principles calculations for pristine $2H$-NbS_2, $2H$-TaS_2, and the Cr-intercalated analogs of these host lattices were performed by using the open source plane-wave code Quantum Espresso (QE).[2] The optimized norm-conserving Vanderbilt (ONCV) pseudopotentials from the PseudoDojo project[3,4] were applied. The scalar relativistic pseudopotentials were used for the collinear spin polarization calculations, and the full relativistic pseudopotentials were used for the noncollinear spin polarization calculations with spin–orbit coupling. The kinetic energy cut-off for wavefunctions were set to 86 Ry for all the self-consistent calculations; for these calculations, the experimental lattice constants obtained from X-ray diffraction were used. Nevertheless, the band structure with the fully optimized structure is similar to that with the experimental lattice constants.[5,6] DFT-D2 van der Waals correction was applied to account for the long-range interaction between layers.[7,8] A Γ-centered $4 \times 4 \times 2$ k-mesh was sampled in the Brillouin zone for both $Cr_{1/3}NbS_2$ and $Cr_{1/3}TaS_2$, and a $8 \times 8 \times 2$ k-mesh for both $2H$-NbS_2 and $2H$-TaS_2. The Perdew–Burke–Ernzerhof (PBE) exchange-correlation functional[9] was used in the spin-polarized calculations. Previous first-principles studies are performed both at the PBE[5,10,11] and at the GGA+U level[6] with an on-site Coulomb interaction, U, of 4 eV for Cr. In this work, different U parameters for Cr, Nb, and Ta were explored. The results obtained from PBE are shown and compared to the experiments in the main text. Calculations obtained for the spin-polarized, orbital-projected, k_z-dependent band structures, and the Fermi surfaces are shown in comparison with the results. In addition, Bader charges were calculated using the Bader charge analysis method.[12–15] Finally, magnetic coupling constants were computed from first-principles at FFM configurations, and Curie temperatures T_C of Cr-intercalated systems were estimated.

U Parameter for Band Structure from GGA+U Calculations

Cr, Nb, and Ta are transition metals for which the U parameter can be used to describe the on-site Coulomb interaction between localized d electrons.[16,17] However, whether or not the

U parameters for the aforementioned transition metals are important for the band structure calculations of $Cr_{1/3}NbS_2$ and $Cr_{1/3}TaS_2$ is not clear. More investigations are necessary beyond the previous studies using PBE[5,10,11] and GGA+U with a U value of 4 eV for Cr 3d electrons.[6] Here, the on-site Coulomb interaction U parameters for Cr and Nb are tested using the values close to those from Ref. [6, 16–19]. The U parameters are discussed in order to clarify the effect of U parameters on the band structure and the necessity of adopting U parameters.

Looking at the band structures with varying U(Cr) in Figure S5(a), overall the band structures of interest within −1 eV to 0 eV do not change much with U(Cr). Moreover, the band structures near the Fermi level do not show qualitative changes, except for the minor upshift of the bands at Γ with increasing U(Cr). The small change of band structures in this energy range may be explained by the small contribution of Cr d orbitals in projected density of states (PDOS) in Figure S5(c), and that the d electrons near the Fermi level are nearly delocalized and thus not affected by the U parameter strongly. Likewise, U(Nb) can be found to be not important to the band structures of interest from Figure S5(b). Therefore, the band structures from PBE are shown in the main text and compared with experiments.

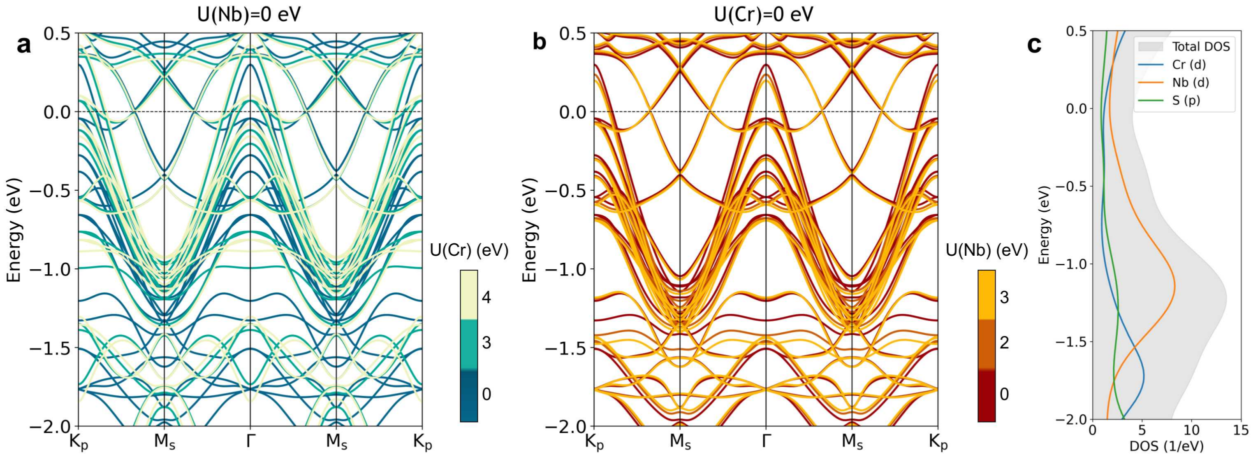

Figure S5: (a) and (b) Band structures of $Cr_{1/3}NbS_2$ with the different on-site Coulomb interaction for Cr and Nb represented as U(Cr) and U(Nb), respectively. (c) Projected density of states of $Cr_{1/3}NbS_2$ calculated with PBE. (a) U(Nb) is set to 0 eV and the effect of U(Cr) on the band structure is shown when varied from 0 to 4 eV. (b) U(Cr) is set to 0 eV and the effect of U(Nb) on the band structure is shown when varied from 0 to 3 eV.

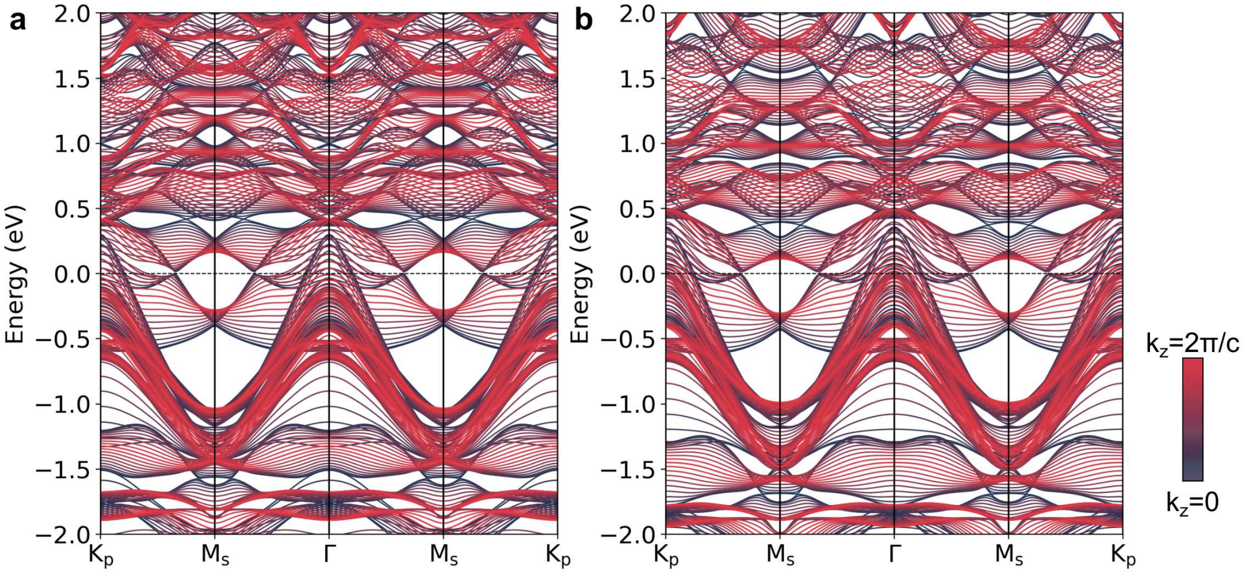

Figure S6: (a) and (b) k_z-projected band structures of $Cr_{1/3}NbS_2$ and $Cr_{1/3}TaS_2$, respectively, calculated using the PBE functional.

4.1 Bader Charge Analysis

The Cr–host lattice hybridization in the vicinity of Fermi level is revealed by the polarization-dependent ARPES and orbital-projected DFT calculations. To estimate the extent to which Cr is hybridized with the host lattice, we evaluated charge transfer between Cr and the host by calculating Bader charge. Comparing the Bader charge in Table S4, we can find the charge transfer of 1.19 electrons from each Cr atom to the host NbS_2 or TaS_2. As a result of charge transfer, Cr becomes positively charged, Nb is less positive, and S is more negative in $Cr_{1/3}NbS_2$ compared to the pristine $2H$-NbS_2. Likewise, the Bader charge of Cr in $Cr_{1/3}TaS_2$ is 1.19 in Table S5, similar to Cr in $Cr_{1/3}NbS_2$. This is consistent with the similarity between the bandstructures of $Cr_{1/3}NbS_2$ and $Cr_{1/3}TaS_2$, and consistent with the similar magnetic moments between $Cr_{1/3}NbS_2$ and $Cr_{1/3}TaS_2$, as shown in Table S6 and S7. Therefore, we conclude that the Cr–host lattice hybridization is similar between $Cr_{1/3}NbS_2$ and $Cr_{1/3}TaS_2$. Figure S7 shows the charge transfer density distribution of $Cr_{1/3}NbS_2$ and $Cr_{1/3}TaS_2$.

Table S4: Bader charge of $2H$-NbS_2 and $Cr_{1/3}NbS_2$. "–" means no value. Nb_1 denotes the nearest Nb atoms to Cr, and Nb_2 denotes the second nearest Nb atoms to Cr. Positive value means electron loss, and negative means electron gain.

Atom	$2H$-NbS_2	$Cr_{1/3}NbS_2$
Cr	–	1.19
Nb_1	1.51	1.37
Nb_2	1.51	1.41
S	−0.76	−0.90

Table S5: Bader charge of $2H$-TaS_2 and $Cr_{1/3}TaS_2$. "–" means no value. Ta_1 denotes the nearest Ta atoms to Cr, and Ta_2 denotes the second nearest Ta atoms to Cr. Positive value means electron loss, and negative means electron gain.

Atom	$2H$-TaS_2	$Cr_{1/3}TaS_2$
Cr	–	1.19
Ta_1	1.79	1.50
Ta_2	1.79	1.59
S	−0.90	−0.98

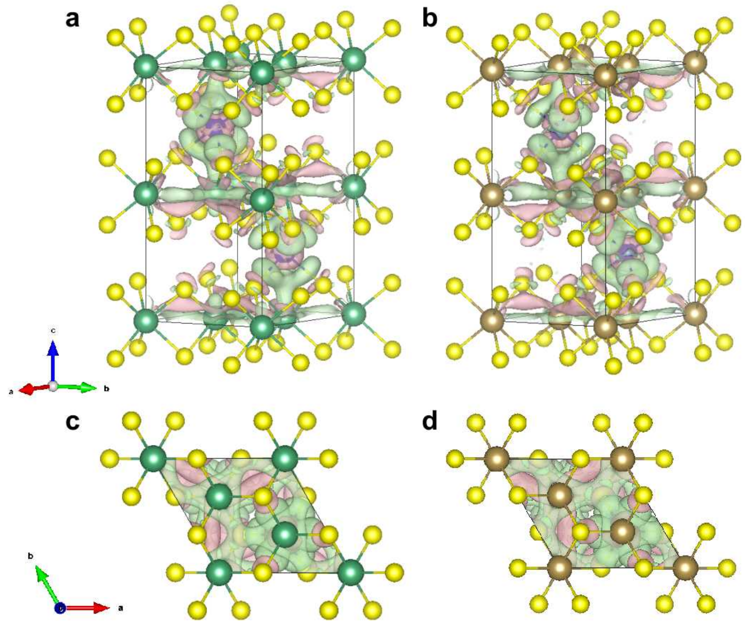

Figure S7: Charge transfer of $Cr_{1/3}NbS_2$ and $Cr_{1/3}TaS_2$. The isosurface is set to 2% of the maximum charge density. The green charge density denotes electron gain, and the pink charge density denotes electron loss. Representations are shown for $Cr_{1/3}NbS_2$ and $Cr_{1/3}TaS_2$ along the crystallographic a-axis in (a) and (b), and crystallographic c-axis in (c) and (d).

4.2 Magnetic Moment

Table S6: Magnetic moment of $Cr_{1/3}NbS_2$.

Stoichiometry	Saturation magnetization (μ_B)	Reference	Notes
1/3	2.9	[20]	$M(H)$ (4.2 K)
1/3	3.89	[21]	$M(H)$ (2 K)
1/3	3.2	[5]	$M(H)$ (2 K)
0.33(1)	2.68	This work	$M(H)$ (2 K)
1/3	2.66	This work	DFT (PBE)

Table S7: Magnetic moment of $Cr_{1/3}TaS_2$.

Stoichiometry	Saturation magnetization (μ_B)	Reference	Notes
1/3	2.97	[22]	$M(H)$ (2 K)
1/3	2.73	[23]	$M(H)$ (2 K)
1/3	2.73	[24]	$M(H)$ (2 K)
0.33(1)	2.82	This work	$M(H)$ (2 K)
1/3	2.71	This work	DFT (PBE)

4.3 Exchange Interaction Analysis

The exchange interaction in $Cr_{1/3}NbS_2$ and $Cr_{1/3}TaS_2$ was modeled using the following Hamiltonian after Ref. [25]:

$$\hat{H} = E_0 + \frac{9}{8}\left(J_1 \sum_{\langle i,j \rangle} \hat{\mathbf{S}}_i \cdot \hat{\mathbf{S}}_j + J_2 \sum_{\langle \tilde{i},\tilde{j} \rangle} \hat{\mathbf{S}}_{\tilde{i}} \cdot \hat{\mathbf{S}}_{\tilde{j}} + J_{1c} \sum_{\langle i_c,j_c \rangle} \hat{\mathbf{S}}_{i_c} \cdot \hat{\mathbf{S}}_{j_c} + J_{2c} \sum_{\langle \tilde{i}_c,\tilde{j}_c \rangle} \hat{\mathbf{S}}_{\tilde{i}_c} \cdot \hat{\mathbf{S}}_{\tilde{j}_c} \right) \quad \text{(S1)}$$

where E_0 contains the nonmagnetic contribution to the total energy, $S = 3/2$ is the total spin of Cr^{3+}, i, j run over all the nearest neighbors in the same plane, $\tilde{i}, \tilde{j}$ run over all the nearest neighbors in the same plane, i_c, j_c run over all the nearest neighbors between two planes, and $\tilde{i}_c, \tilde{j}_c$ run over all the second nearest neighbors between two planes. Because the Cr–Cr distance for J_2 is as large as 9.9 Å, the interaction is expected to be small like $Fe_{1/3}NbS_2$;[25] therefore, we ignore J_2. Then, J_1, J_{1c} and J_{1c} are considered and arranged in the ascending order of Cr–Cr distance.

Because the total spin of Cr^{3+} is large, we can treat the model classically in the Ising limit.[25] And following the previous studies,[25,26] we take eight spin configurations into consideration, and they are sufficient for the evaluation of the exchange interaction constants. Figure S8 shows the out-of-plane antiferromagnetic (AFM) configuration as an example. The energy of the configurations are summarized in Table S8 and Table S9 for $Cr_{1/3}NbS_2$ and $Cr_{1/3}TaS_2$, respectively. The combination of any configurations that gives rise to zero magnetic contribution to the total energy is set as the zero reference for all configurations. In this study, the total energy reference is taken as $\mathrm{E_{ref}} = (\mathrm{E_{FM-AFM1}} + \mathrm{E_{AFM4}})/2$. Then the exchange interaction constants are evaluated by a least squares fitting to the total energy of all the considered configurations. The results can be found in Table S10 and Table S11 for $Cr_{1/3}NbS_2$ and $Cr_{1/3}TaS_2$, respectively. The coefficient of determination is also provided in Figure S9 to show the excellent fitting quality.

We roughly estimate the overall coupling strength $J = 6(J_1 + J_{1c} + J_{2c})$ with considering that each Cr has six nearest neighbors and that J, J_{1c} and J_{2c} are of equal weight, yielding

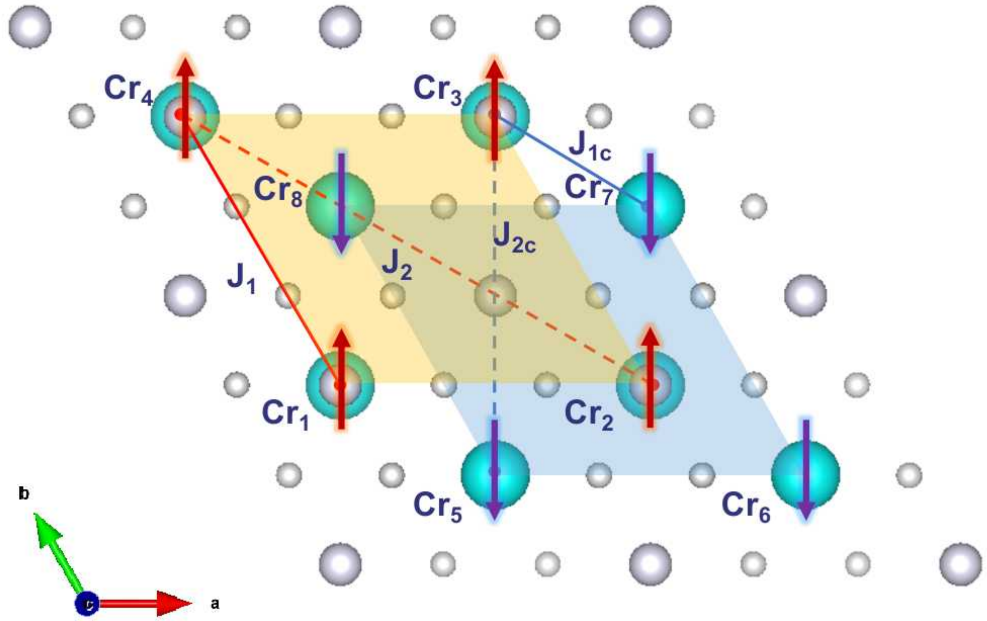

Figure S8: Schematic diagram of a antiferromagnetic (AFM) spin configuration layout along the crystallographic c-axis. Blue atoms denote Cr, large gray atoms denote Nb (Ta), and small gray atoms denote S. Eight Cr atoms in one cell are labeled from 1 to 8, with 1–4 being on one plane and 5–8 being on the other plane. Each Cr atom has either spin up (red arrow) or spin down (purple arrow). The yellow plane and blue plane are the mutually nearest planes in the same cell. J_1 and J_2 are the exchange interactions between the nearest neighbors and the second nearest neighbors on the same plane, respectively. They are separately labeled by the red solid line and red dashed line. Similarly, J_{1c} and J_{2c} are the exchange interactions between the nearest neighbors and the second nearest neighbors between the nearest planes, respectively. They are separately labeled by the blue solid line and blue dashed line.

Table S8: Energy of different spin configurations of $Cr_{1/3}NbS_2$ (with $E_{ref} = (E_{FM-AFM1} + E_{AFM4})/2$ as the energy reference zero). The notation $|s_1 s_2 s_3 s_4 s_5 s_6 s_7 s_8\rangle$ is adopted to denote each of the configurations, with s_i meaning the spin (either spin up $|\uparrow\rangle$ or spin down $|\downarrow\rangle$) of the i-th Cr site as can be seen in Figure S8.

Configuration	Magnetization Type	Exchange Energy Expression	Energy (meV)
$\lvert\uparrow\uparrow\uparrow\uparrow\uparrow\uparrow\uparrow\uparrow\rangle$	FM	$54J_1 + 54J_{1c} + 54J_{2c}$	-16.45
$\lvert\uparrow\uparrow\uparrow\uparrow\downarrow\downarrow\downarrow\downarrow\rangle$	AFM1	$54J_1 - 54J_{1c} - 54J_{2c}$	7.67
$\lvert\uparrow\downarrow\downarrow\uparrow\uparrow\downarrow\downarrow\uparrow\rangle$	AFM2	$-18J_1 + 18J_{1c} - 54J_{2c}$	2.77
$\lvert\uparrow\uparrow\uparrow\uparrow\uparrow\downarrow\downarrow\uparrow\rangle$	FM-AFM1	$18J_1$	-1.48
$\lvert\uparrow\uparrow\downarrow\uparrow\uparrow\uparrow\downarrow\uparrow\rangle$	FM-AFM2	$18J_{1c}$	-3.20
$\lvert\downarrow\uparrow\uparrow\downarrow\uparrow\downarrow\downarrow\uparrow\rangle$	AFM3	$-18J_1 + 18J_{1c} + 54J_{2c}$	2.77
$\lvert\uparrow\uparrow\downarrow\uparrow\downarrow\uparrow\uparrow\uparrow\rangle$	FM-AFM2	$24J_{2c}$	-1.95
$\lvert\downarrow\uparrow\uparrow\downarrow\uparrow\uparrow\downarrow\downarrow\rangle$	AFM4	$-18J_1$	1.48

Table S9: Energy of different spin configurations of $Cr_{1/3}TaS_2$ (with $E_{ref} = (E_{FM-AFM1} + E_{AFM4})/2$ as the energy reference zero). The notation $|s_1 s_2 s_3 s_4 s_5 s_6 s_7 s_8\rangle$ is adopted to denote each of the configurations, with s_i meaning the spin (either spin up $|\uparrow\rangle$ or spin down $|\downarrow\rangle$) of the i-th Cr site as can be seen in Figure S8.

Configuration	Magnetization Type	Exchange Energy Expression	Energy (meV)
$\lvert\uparrow\uparrow\uparrow\uparrow\uparrow\uparrow\uparrow\uparrow\rangle$	FM	$54J_1 + 54J_{1c} + 54J_{2c}$	-17.61
$\lvert\uparrow\uparrow\uparrow\uparrow\downarrow\downarrow\downarrow\downarrow\rangle$	AFM1	$54J_1 - 54J_{1c} - 54J_{2c}$	10.70
$\lvert\uparrow\downarrow\downarrow\uparrow\uparrow\downarrow\downarrow\uparrow\rangle$	AFM2	$-18J_1 + 18J_{1c} - 54J_{2c}$	2.63
$\lvert\uparrow\uparrow\uparrow\uparrow\uparrow\downarrow\downarrow\uparrow\rangle$	FM-AFM1	$18J_1$	-1.14
$\lvert\uparrow\uparrow\downarrow\uparrow\uparrow\uparrow\downarrow\uparrow\rangle$	FM-AFM2	$18J_{1c}$	-3.75
$\lvert\downarrow\uparrow\uparrow\downarrow\uparrow\downarrow\downarrow\uparrow\rangle$	AFM3	$-18J_1 + 18J_{1c} + 54J_{2c}$	2.63
$\lvert\uparrow\uparrow\downarrow\uparrow\downarrow\uparrow\uparrow\uparrow\rangle$	FM-AFM2	$24J_{2c}$	-2.42
$\lvert\downarrow\uparrow\uparrow\downarrow\uparrow\uparrow\downarrow\downarrow\rangle$	AFM4	$-18J_1$	1.14

$J = -3.72$ meV for $Cr_{1/3}NbS_2$ and $J = -3.96$ meV for $Cr_{1/3}TaS_2$. Furthermore, using these estimated J and the equation of Curie temperature under molecular-field approximation,[27]

$$T_C = 2JS(S+1)/3k_B \tag{S2}$$

where k_B is the Boltzmann constant, with $S = 3/2$ being the avarage spin on each Cr site. We estimate T_C values of 109 K for $Cr_{1/3}NbS_2$ and 117 K for $Cr_{1/3}TaS_2$, which are reasonably close to the previously reported experimental values 127 K and 120 K of $Cr_{1/3}NbS_2$[5,28] and 146 K and 150 K of $Cr_{1/3}TaS_2$,[24,29] and in line with the T_C values reported in this work.

Finally, the FM configuration, which is stablized by applying an external magnetic field in experiments, was adopted in our DFT calculations earlier. The applied magnetic field in experiments can cause energy change due to Zeeman effect, and the energy change may need

Table S10: Fitting results for coupling in $Cr_{1/3}NbS_2$.

Exchange	No. Neighbors	Cr-Cr distance (Å)	Value (meV)
J_1	6	5.74	-0.18
J_{1c}	6	6.90	-0.33
J_{2c}	6	8.98	-0.11

Table S11: Fitting results for coupling in $Cr_{1/3}TaS_2$.

Exchange	No. Neighbors	Cr-Cr distance (Å)	Value (meV)
J_1	6	5.71	-0.14
J_{1c}	6	6.92	-0.39
J_{2c}	6	8.97	-0.13

to be considered when fitting the exchange interaction constants. To explain the effect of the external magnetic field, we substract the Zeeman term from the Hamiltonian in Eq. S1

$$\hat{H} = E_0 + \frac{9}{8}\left(J_1 \sum_{\langle i,j \rangle} \hat{\mathbf{S}}_i \cdot \hat{\mathbf{S}}_j + J_2 \sum_{\langle \tilde{i},\tilde{j} \rangle} \hat{\mathbf{S}}_{\tilde{i}} \cdot \hat{\mathbf{S}}_{\tilde{j}} + J_{1c} \sum_{\langle i_c,j_c \rangle} \hat{\mathbf{S}}_{i_c} \cdot \hat{\mathbf{S}}_{j_c} + J_{2c} \sum_{\langle \tilde{i}_c,\tilde{j}_c \rangle} \hat{\mathbf{S}}_{\tilde{i}_c} \cdot \hat{\mathbf{S}}_{\tilde{j}_c} \right) - g\mu_B \mathbf{B} \cdot \sum_i \hat{\mathbf{S}}_i \tag{S3}$$

where g factor is estimated to be 2,[30] and $\mathbf{B}$ is the magnetic field whose range is set to cover the critical field ~0.15 T of $Cr_{1/3}NbS_2$[5] and ~1.5 T of $Cr_{1/3}TaS_2$.[22] J_1, J_{1c} and J_{2c} are enhanced linearly with increasing the magnetic field, so as for the calculated Curie temperature. The result shows that whether or not the Zeeman effect is taken into consideration does not alter the conclusion that the fitting result agrees with the experiments. Most importantly, the calculated Curie temperature (or the total J) is always higher in $Cr_{1/3}TaS_2$ than $Cr_{1/3}NbS_2$ under the same $\mathbf{B}$ field, consistent with the experimental observation. This indicates that the FM configuration is valid for the DFT calculations.

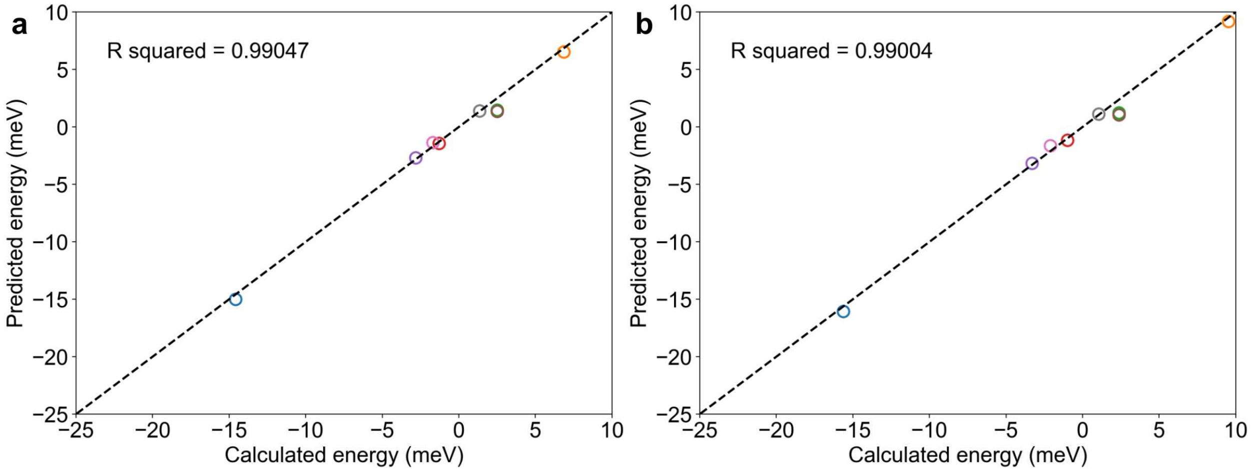

Figure S9: Comparison of the predicted total energy using the spin Hamiltonian Eq. S1 and the fitted exchange interaction constants J_1, J_{1c} and J_{2c}, with the calculated total energy, for (a) $Cr_{1/3}NbS_2$ and (b) $Cr_{1/3}TaS_2$.

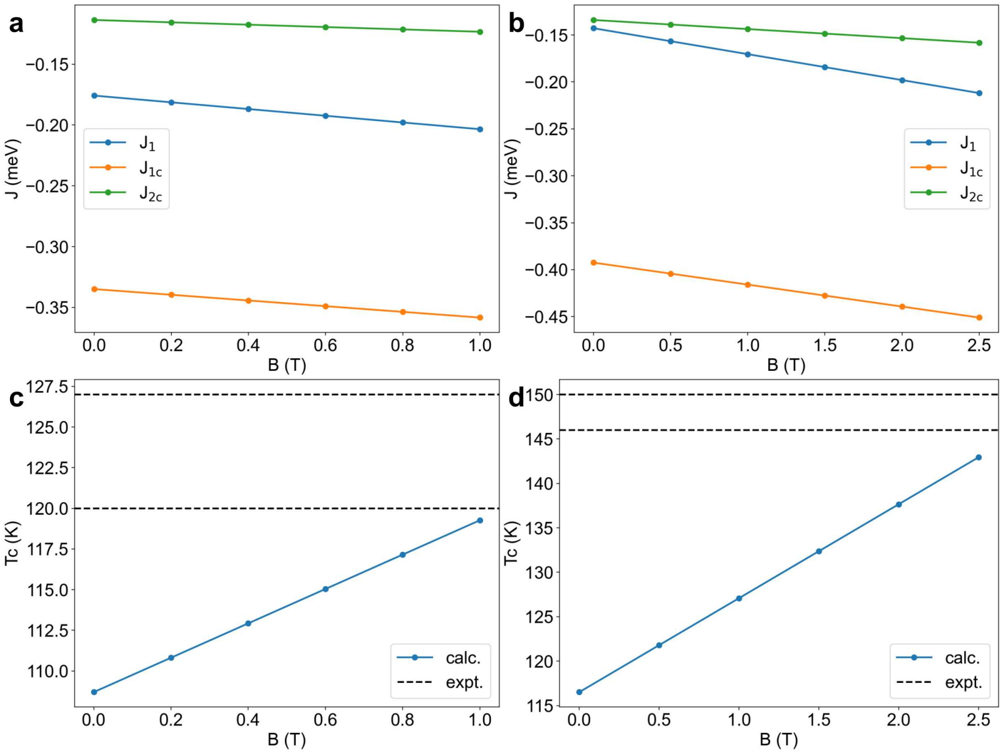

Figure S10: The exchange interaction constants J_1, J_{1c} and J_{2c} of (a) $Cr_{1/3}NbS_2$ and (b) $Cr_{1/3}TaS_2$, and the Curie temperature of (c) $Cr_{1/3}NbS_2$ and (d) $Cr_{1/3}TaS_2$ against the external magnetic field **B**. The horizontal dashed lines in (c) and (d) denotes the experimental Curie temperatures.[5,24,28,29].

4.4 Effect of Spin–Orbit Coupling on Band Structure

To evaluate the effect of spin–orbit coupling (SOC) on band structures of $Cr_{1/3}NbS_2$ and $Cr_{1/3}TaS_2$, we compare the band structures with and without SOC for each system in Figure S11. The comparison shows that the band structure of $Cr_{1/3}NbS_2$ without SOC nearly

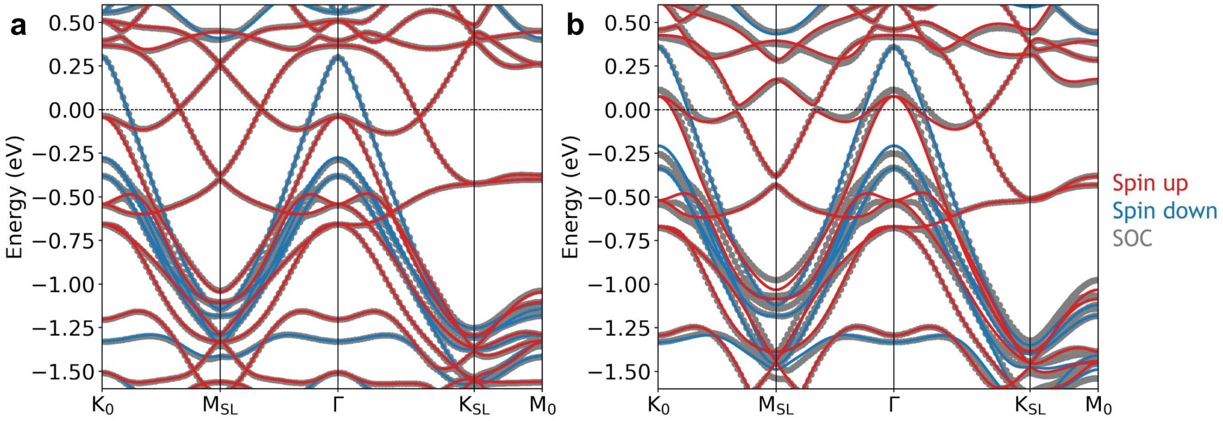

Figure S11: Band structures of (a) $Cr_{1/3}NbS_2$ and (b) $Cr_{1/3}TaS_2$. The band structures with spin up (red) and spin down (blue) are from the collinear spin-polarized calculations without SOC, and the band structure in gray are from the noncollinear spin-polarized calculations with SOC.

overlaps with the band structure with SOC in the energy range of interest (−1 eV to 0 eV), and the band structure of $Cr_{1/3}TaS_2$ without SOC is qualitatively similar (eigenenergy difference < 0.05 eV) to the band structure with SOC in the energy range of interest. The small difference can be neglected when comparing the calculated band structure to the experimental band structure. In addition, Ref. [5, 6] also explain that the collinear spin polarization is adequate for the band structure calculation of $Cr_{1/3}NbS_2$. Therefore, the result supports that SOC effect does not change much of band structure and supports the choice of collinear spin polarization in our first-principles calculations.

References

(1) Fan, S.; Neal, S.; Won, C.; Kim, J.; Sapkota, D.; Huang, F.; Yang, J.; Mandrus, D. G.; Cheong, S.-W.; Haraldsen, J. T.; Musfeldt, J. L. Excitations of Intercalated Metal Monolayers in Transition Metal Dichalcogenides. *Nano Lett.* **2021**, *21*, 99–106.

(2) Giannozzi, P.; Baroni, S.; Bonini, N.; Calandra, M.; Car, R.; Cavazzoni, C.; Ceresoli, D.; Chiarotti, G. L.; Cococcioni, M.; Dabo, I.; Dal Corso, A.; de Gironcoli, S.; Fabris, S.; Fratesi, G.; Gebauer, R.; Gerstmann, U.; Gougoussis, C.; Kokalj, A.; Lazzeri, M.; Martin-Samos, L.; Marzari, N.; Mauri, F.; Mazzarello, R.; Paolini, S.; Pasquarello, A.; Paulatto, L.; Sbraccia, C.; Scandolo, S.; Sclauzero, G.; Seitsonen, A. P.; Smogunov, A.; Umari, P.; Wentzcovitch, R. M. QUANTUM ESPRESSO: A Modular and Open-Source Software Project for Quantum Simulations of Materials. *J. Phys.: Condens. Matter* **2009**, *21*, 395502.

(3) Hamann, D. R. Optimized Norm-Conserving Vanderbilt Pseudopotentials. *Phys. Rev. B* **2013**, *88*, 085117.

(4) Van Setten, M. J.; Giantomassi, M.; Bousquet, E.; Verstraete, M. J.; Hamann, D. R.; Gonze, X.; Rignanese, G.-M. The PseudoDojo: Training and grading a 85 element optimized norm-conserving pseudopotential table. *Comput. Phys. Commun.* **2018**, *226*, 39–54.

(5) Ghimire, N. J.; McGuire, M. A.; Parker, D. S.; Sipos, B.; Tang, S.; Yan, J.-Q.; Sales, B. C.; Mandrus, D. Magnetic Phase Transition in Single Crystals of the Chiral Helimagnet $Cr_{1/3}NbS_2$. *Phys. Rev. B* **2013**, *87*, 104403.

(6) Qin, N.; Chen, C.; Du, S.; Du, X.; Zhang, X.; Yin, Z.; Zhou, J.; Xu, R.; Gu, X.; Zhang, Q.; Zhao, W.; Li, Y.; Mo, S.-K.; Liu, Z.; Zhang, S.; Guo, Y.; Tang, P.; Chen, Y.; Yang, L. Persistent Exchange Splitting in the Chiral Helimagnet $Cr_{1/3}NbS_2$. *Phys. Rev. B* **2022**, *106*, 035129.

(7) Grimme, S. Semiempirical GGA-type density functional constructed with a long-range dispersion correction. *J. Comput. Chem.* **2006**, *27*, 1787–1799.

(8) Barone, V.; Casarin, M.; Forrer, D.; Pavone, M.; Sambi, M.; Vittadini, A. Role and effective treatment of dispersive forces in materials: Polyethylene and graphite crystals as test cases. *J. Comput. Chem.* **2009**, *30*, 934–939.

(9) Perdew, J. P.; Burke, K.; Ernzerhof, M. Generalized Gradient Approximation Made Simple. *Phys. Rev. Lett.* **1996**, *77*, 3865.

(10) Bornstein, A. C.; Chapman, B. J.; Ghimire, N. J.; Mandrus, D. G.; Parker, D. S.; Lee, M. Out-of-Plane Spin-Orientation Dependent Magnetotransport Properties in the Anisotropic Helimagnet $Cr_{1/3}NbS_2$. *Phys. Rev. B* **2015**, *91*, 184401.

(11) Sirica, N.; Hedayat, H.; Bugini, D.; Koehler, M. R.; Li, L.; Parker, D. S.; Mandrus, D. G.; Dallera, C.; Carpene, E.; Mannella, N. Disentangling Electronic, Lattice, and Spin Dynamics in the Chiral Helimagnet $Cr_{1/3}NbS_2$. *Phys. Rev. B* **2021**, *104*, 174426.

(12) Tang, W.; Sanville, E.; Henkelman, G. A grid-based Bader analysis algorithm without lattice bias. *J. Phys. Condens. Matter* **2009**, *21*, 084204.

(13) Sanville, E.; Kenny, S. D.; Smith, R.; Henkelman, G. Improved grid-based algorithm for Bader charge allocation. *J. Comput. Chem.* **2007**, *28*, 899–908.

(14) Henkelman, G.; Arnaldsson, A.; Jónsson, H. A fast and robust algorithm for Bader decomposition of charge density. *Comput. Mater. Sci.* **2006**, *36*, 354–360.

(15) Yu, M.; Trinkle, D. R. Accurate and efficient algorithm for Bader charge integration. *J. Chem. Phys.* **2011**, *134*.

(16) Shi, S.; Wysocki, A. L.; Belashchenko, K. D. Magnetism of chromia from first-principles calculations. *Phys. Rev. B* **2009**, *79*, 104404.

(17) Şaşıoğlu, E.; Friedrich, C.; Blügel, S. Effective Coulomb interaction in transition metals from constrained random-phase approximation. *Phys. Rev. B* **2011**, *83*, 121101.

(18) Pasquier, D.; Yazyev, O. V. Charge density wave phase, Mottness, and ferromagnetism in monolayer $1T$-$NbSe_2$. *Phys. Rev. B* **2018**, *98*, 045114.

(19) Calandra, M. Phonon-Assisted Magnetic Mott-Insulating State in the Charge Density Wave Phase of Single-Layer $1T$-$NbSe_2$. *Phys. Rev. Lett.* **2018**, *121*, 026401.

(20) Miyadai, T.; Kikuchi, K.; Kondo, H.; Sakka, S.; Arai, M.; Ishikawa, Y. Magnetic Properties of $Cr_{1/3}NbS_2$. *J. Phys. Soc. Jpn.* **1983**, *52*, 1394–1401.

(21) Hulliger, F.; Pobitschka, E. On the magnetic behavior of new 2*H*-NbS_2-type derivatives. *J. Solid State Chem.* **1970**, *1*, 117–119.

(22) Zhang, C.; Zhang, J.; Liu, C.; Zhang, S.; Yuan, Y.; Li, P.; Wen, Y.; Jiang, Z.; Zhou, B.; Lei, Y.; Zheng, D.; Song, C.; Hou, Z.; Mi, W.; Schwingenschlögl, U.; Manchon, A.; Qiu, Z. Q.; Alshareef, H. N.; Peng, Y.; Zhang, X.-X. Chiral Helimagnetism and One-Dimensional Magnetic Solitons in a Cr-Intercalated Transition Metal Dichalcogenide. *Adv. Mater.* **2021**, *33*, 2101131.

(23) Obeysekera, D.; Gamage, K.; Gao, Y.; Cheong, S.-w.; Yang, J. The Magneto-Transport Properties of $Cr_{1/3}TaS_2$ with Chiral Magnetic Solitons. *Adv. Electron. Mater.* **2021**, *7*, 2100424.

(24) Du, K.; Huang, F.-T.; Kim, J.; Lim, S. J.; Gamage, K.; Yang, J.; Mostovoy, M.; Garlow, J.; Han, M.-G.; Zhu, Y.; Cheong, S.-W. Topological Spin/Structure Couplings in Layered Chiral Magnet $Cr_{1/3}TaS_2$: The Discovery of Spiral Magnetic Superstructure. *Proc. Natl. Acad. Sci.* **2021**, *118*, e2023337118.

(25) Haley, S. C.; Weber, S. F.; Cookmeyer, T.; Parker, D. E.; Maniv, E.; Maksimovic, N.; John, C.; Doyle, S.; Maniv, A.; Ramakrishna, S. K.; Reyes, A. P.; Singleton, J.; Moore, J. E.; Neaton, J. B.; Analytis, J. G. Half-magnetization plateau and the origin of threefold symmetry breaking in an electrically switchable triangular antiferromagnet. *Phys. Rev. Res.* **2020**, *2*, 043020.

(26) Smart, T. J.; Cardiel, A. C.; Wu, F.; Choi, K.-S.; Ping, Y. Mechanistic insights of enhanced spin polaron conduction in CuO through atomic doping. *npj Comput. Mater.* **2018**, *4*, 61.

(27) Yosida, K., *Theory of magnetism*; Springer Science & Business Media: 1996; Vol. 122.

(28) Togawa, Y.; Koyama, T.; Takayanagi, K.; Mori, S.; Kousaka, Y.; Akimitsu, J.; Nishihara, S.; Inoue, K.; Ovchinnikov, A. S.; Kishine, J. Chiral Magnetic Soliton Lattice on a Chiral Helimagnet. *Phys. Rev. Lett.* **2012**, *108*, 107202.

(29) Meng, F.; Liu, W.; Rahman, A.; Zhang, J.; Fan, J.; Ma, C.; Ge, M.; Yao, T.; Pi, L.; Zhang, L.; Zhang, Y. Crossover of critical behavior and nontrivial magnetism in the chiral soliton lattice host $Cr_{1/3}TaS_2$. *Phys. Rev. B* **2023**, *107*, 144425.

(30) Wan-Lun, Y.; Min-Guang, Z. Effects of high pressure on the g-factors of Cr3+: MgO. *J. Phys. C: Solid State Phys.* **1987**, *20*, 2923.
